# Acinetobacter baumannii Catabolizes Ethanolamine in the Absence of a Metabolosome and Converts Cobinamide into Adenosylated Cobamides

**DOI:** 10.1128/mbio.01793-22

**Published:** 2022-07-26

**Authors:** Elizabeth A. Villa, Jorge C. Escalante-Semerena

**Affiliations:** a Department of Microbiology, University of Georgia, Athens, Georgia, USA; University of Michigan—Ann Arbor

**Keywords:** *Acinetobacter baumannii*, ethanolamine catabolism, ethanolamine ammonia-lyase, cobinamide salvaging, metabolosome, bacterial microcompartments, nucleotide loop assembly, B_12_ biosynthesis, cobamide, ethanolamine ammonia-lyase reactivation, metabolosomes

## Abstract

Acinetobacter baumannii is an opportunistic pathogen typically associated with hospital-acquired infections. Our understanding of the metabolism and physiology of A. baumannii is limited. Here, we report that A. baumannii uses ethanolamine (EA) as the sole source of nitrogen and can use this aminoalcohol as a source of carbon and energy if the expression of the *eutBC* genes encoding ethanolamine ammonia-lyase (EAL) is increased. A strain with an IS*Aba1* element upstream of the *eutBC* genes efficiently used EA as a carbon and energy source. The A. baumannii EAL (*Ab*EAL) enzyme supported the growth of a strain of Salmonella lacking the entire *eut* operon. Remarkably, the growth of the above-mentioned Salmonella strain did not require the metabolosome, the reactivase EutA enzyme, the EutE acetaldehyde dehydrogenase, or the addition of glutathione to the medium. Transmission electron micrographs showed that when Acinetobacter baumannii or Salmonella enterica subsp. *enterica* serovar Typhimurium strain LT2 synthesized *Ab*EAL, the protein localized to the cell membrane. We also report that the A. baumannii genome encodes all of the enzymes needed for the assembly of the nucleotide loop of cobamides and that it uses these enzymes to synthesize different cobamides from the precursor cobinamide and several nucleobases. In the absence of exogenous nucleobases, the most abundant cobamide produced by A. baumannii was cobalamin.

## INTRODUCTION

Acinetobacter baumannii is a nonmotile Gram-negative bacillus of concern to public health due to its ability to rapidly develop resistance to antibiotics, leading to multidrug-resistant strains that are difficult to treat. At present, A. baumannii is listed as a high-priority pathogen for research and development of new antibiotics by the World Health Organization.

A goal of this study was to determine whether A. baumannii could catabolize ethanolamine (EA) and, if so, which enzymes were required to degrade this aminoalcohol. Inspection of the genome of this bacterium suggested that EA could be catabolized and used as a source of carbon, energy, and nitrogen. In pathogens such as Salmonella enterica subsp. *enterica* serovar Typhimurium strain LT2 (hereafter *S.* Typhimurium), EA catabolism plays an important role in cell fitness, survival, and infection (1–3).

**FIG 1:**
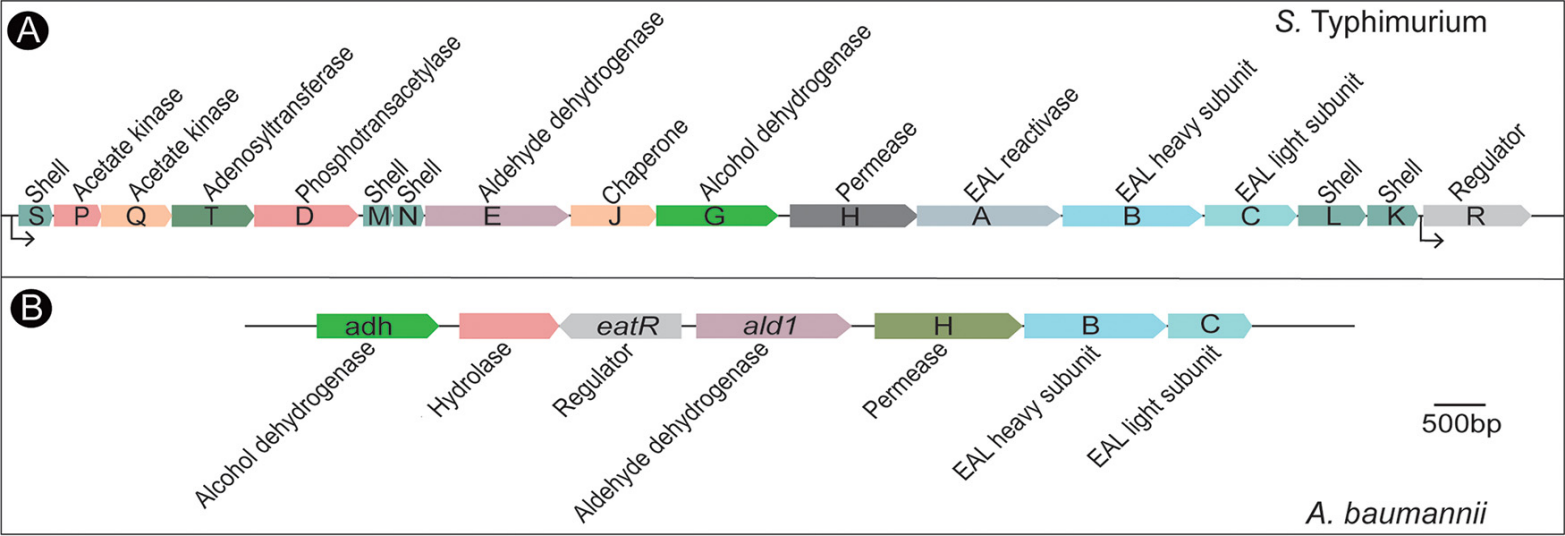
Comparison of the *eut* genes in *S.* Typhimurium and A. baumannii. (A) In *S.* Typhimurium, a total of 17 gene functions are needed for ethanolamine utilization (*eut* genes) as a carbon, nitrogen, and energy source under certain growth conditions. (B) In contrast, A. baumannii may require seven or fewer putative *eut* genes to break down ethanolamine. Genes are at the scale indicated by the bar.

Bioinformatics analysis of the A. baumannii genome provided a very intriguing picture of how EA may be catabolized in this bacterium. We identified homologues of some of the genes required for EA degradation in *S.* Typhimurium, where such metabolic capabilities have been studied in detail (4). The genome of *S.* Typhimurium contains a 16-gene operon (*eutSPQTDMNEJGHABCLK*) that encodes functions needed for the safe breakdown of EA inside a structure that is referred to as the Eut metabolosome (5, 6). *S.* Typhimurium controls the expression of the *eut* operon with an AraC-like protein known as EutR, which binds adenosylcobalamin (AdoCbl) and EA before it activates the expression of the *eut* operon (Fig. 1A). In contrast, the A. baumannii genome encodes only a putative 4-gene *eut* operon (locus tags A1S_2102, A1S_2103, A1S_2104, and A1S_2106) and a divergently transcribed regulator (*eatR* [locus tag A1S_2101]) encoding a σ^54^ enhancer binding protein (7) (Fig. 1B). Two additional genes immediately upstream and divergently transcribed from the putative regulator may also participate in the catabolism of EA. If this were the case, six functions would be needed to break down EA in A. baumannii (Fig. 1B; see also Fig. S1 in the supplemental material). Notably, the genome does not encode the shell proteins that comprise the *S.* Typhimurium metabolosome. This fact is important because it suggests that EA catabolism in A. baumannii is not contained within a metabolosome, raising the question of how this bacterium controls reactive intermediates generated during EA catabolism in the absence of such a physical structure.

10.1128/mbio.01793-22.1 FIG S1 Sequence alignment of *eut*-associated proteins from A. baumannii and *S.* Typhimurium. Identical amino acids are highlighted in red, and similar amino acids are boxed in blue. Alignments were generated using Clustal Omega 1.2.3 in Geneious Prime software and ESPript 3 (https://espript.ibcp.fr/ESPript/ESPript/). Download FIG S1, PDF file, 1.3 MB. Copyright © 2022 Villa and Escalante-Semerena. 2022 Villa and Escalante-Semerena. https://creativecommons.org/licenses/by/4.0/ This content is distributed under the terms of the Creative Commons Attribution 4.0 International license.

**FIG 2:**
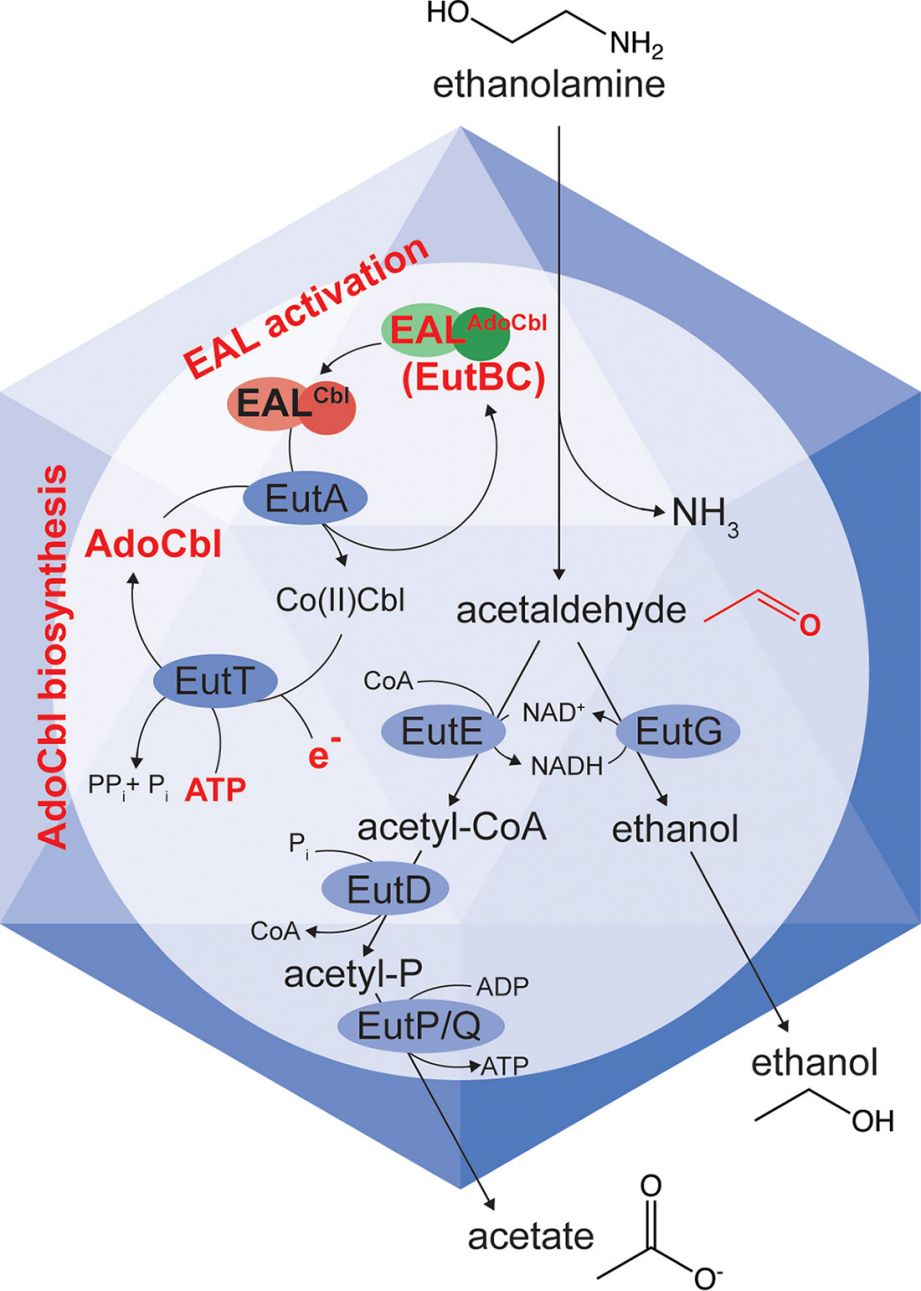
The *S.* Typhimurium Eut metabolosome. Indicated in red are the functions present in A. baumannii. The genome of this bacterium does not encode any of the shell proteins. Highlighted in red are areas of the pathway studied in this work.

The first step of EA catabolism is catalyzed by the AdoCbl-dependent EA ammonia-lyase (EAL) encoded by the *eutBC* genes (8–10) (Fig. 2). As mentioned above, in *S.* Typhimurium, this metabolic pathway occurs inside the metabolosome (6) to prevent cell damage by acetaldehyde and to sequester and concentrate the substrates, enzymes, and coenzymes needed for the efficient catabolism of EA (e.g., AdoCbl, NAD^+^, and CoA) (11, 12). Previous studies in *S.* Typhimurium demonstrated that under certain conditions, EAL alone was sufficient for ethanolamine catabolism only when exogenous glutathione was provided to mitigate the damaging effects of acetaldehyde (6).

Figure 2 depicts two steps pertinent to the work reported here, namely, the conversion of Co(II)Cbl to AdoCbl by EutT (“AdoCbl biosynthesis” in Fig. 2) and the exchange of AdoCbl for Co(II)Cbl by EutA (“EAL activation” in Fig. 2). Notably, the A. baumannii genome lacks a EutT-type ATP:co(I)rrinoid adenosyltransferase (ACAT) (13–17) needed for the synthesis of an adenosylcobamide (AdoCba), the cofactor of the EAL enzyme. We have identified a putative PduO-type ACAT elsewhere on the chromosome (locus tag A1S_2878) (Fig. S2). PduO ACATs are the most widespread ACATs in prokaryotes, and homologues are the only type of ACATs found in humans (18–20).

10.1128/mbio.01793-22.2 FIG S2 A. baumannii encodes a putative ATP:Co (I)rrinoid adenosyltransferase (ACAT). (A) Sequence alignment of A. baumannii AcaT and Lactobacillus reuteri PduO. Identical amino acids are highlighted in red, and similar amino acids are boxed in blue. Alignments were generated using Clustal Omega 1.2.3 in Geneious Prime software and ESPript 3 (https://espript.ibcp.fr/ESPript/ESPript/). (B) Corrinoid adenosylation pathway. As shown by the scheme, the reduction of Co(III) to Co(II) results in a 5-coordinate Co(II) precursor. The second, one-electron reduction that converts Co(II) to Co(I) occurs after binding of the 5-coordinate Co(II) precursor to the ACAT enzyme. As soon as the Co(I) supernucleophile is formed, the attack on the 5′ carbon of the ribose of ATP occurs, displacing triphosphate and yielding the unique Co-C organometallic bond found in adenosylated corrinoids (P. E. Mera and J. C. Escalante-Semerena, Appl Microbiol Biotechnol 88:41–48, 2010, https://doi.org/10.1007/s00253-010-2773-2). Download FIG S2, PDF file, 0.5 MB. Copyright © 2022 Villa and Escalante-Semerena. 2022 Villa and Escalante-Semerena. https://creativecommons.org/licenses/by/4.0/ This content is distributed under the terms of the Creative Commons Attribution 4.0 International license.

The absence of most *eut* genes in A. baumannii suggests that this organism may have evolved a new way of breaking down EA that does not expose the cell to acetaldehyde damage. Furthermore, the lack of an identifiable EutA reactivase structural homologue presents two possibilities: either A. baumannii EAL (*Ab*EAL) better protects AdoCbl from inactivation than *S.* Typhimurium EAL or there is a functional homologue of the *S.* Typhimurium EutA (*Se*EutA) reactivase that is yet to be discovered in A. baumannii.

**FIG 3:**
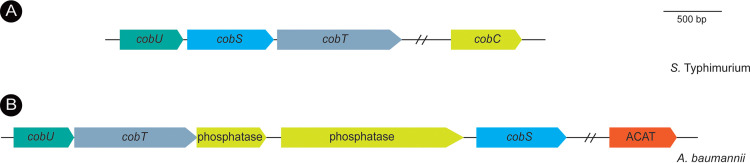
Organization of genes encoding nucleotide loop assembly enzymes in *S.* Typhimurium and A. baumannii. (A) *S.* Typhimurium enzymes have been studied in detail. (B) The A. baumannii genes encode hypothetical proteins whose enzymatic activities had not been described prior to this work. *cobU* encodes the bifunctional nucleotide triphosphate (NTP):Ado-cobinamide kinase (EC 2.7.1.156)–GTP:Adenosylcobinamide phosphate (AdoCbi-P) guanylyltransferase (EC 2.7.7.62), *cobT* encodes nicotinate mononucleotide:dimethylbenzimidazole phosphoribosyltransferase (EC 2.4.2.21), *cobS* encodes AdoCba 5′-P synthase (EC 2.7.8.26), and *cobC* encodes AdoCba 5′-P phosphatase (EC 3.1.3.73). ACAT is the abbreviation for ATP:co(I)rrinoid adenosyltransferase (EC 2.5.1.17).

While the A. baumannii genome does not encode functions for the *de novo* synthesis of the corrin ring of AdoCba, it does encode homologues of enzymes required for the assembly of the nucleotide loop of AdoCba (Fig. 3). These genes are *cobU* (A1S_1659), *cobS* (A1S_1663), *cobT* (A1S_1660), and *cobC* (A1S_1661) (Fig. S3), and studies in other organisms have shown that their products activate the corrin ring and the nucleobase, condense the activated precursors, and dephosphorylate the last intermediate to yield biologically active cobamides (Fig. S4) (21). Collectively, the presence of these putative functions suggested that A. baumannii could salvage incomplete corrinoids and convert them into biologically active coenzymes.

10.1128/mbio.01793-22.3 FIG S3 Sequence alignment of A. baumannii and *S.* Typhimurium NLA enzymes. Identical amino acids are highlighted in red, and similar amino acids are boxed in blue. Alignments were generated using Clustal Omega 1.2.3 in Geneious Prime software and ESPript 3 (https://espript.ibcp.fr/ESPript/ESPript/). Download FIG S3, PDF file, 0.8 MB. Copyright © 2022 Villa and Escalante-Semerena. 2022 Villa and Escalante-Semerena. https://creativecommons.org/licenses/by/4.0/ This content is distributed under the terms of the Creative Commons Attribution 4.0 International license.

10.1128/mbio.01793-22.4 FIG S4 The NLA pathway in *S.* Typhimurium. CobU, a bifunctional NTP:Ado-cobinamide kinase (EC 2.7.1.156) and GTP:AdoCbi-P guanylyltransferase (EC 2.7.7.62), and CobT, a nicotinate mononucleotide:dimethylbenzimidazole phosphoribosyltransferase, generate the substrates for CobS. CobS is an AdoCba 5′-P synthase (EC 2.7.8.26), which condenses these two substrates, and CobC is an AdoCba 5′-P phosphatase (EC 3.1.3.73). Download FIG S4, PDF file, 2.0 MB. Copyright © 2022 Villa and Escalante-Semerena. 2022 Villa and Escalante-Semerena. https://creativecommons.org/licenses/by/4.0/ This content is distributed under the terms of the Creative Commons Attribution 4.0 International license.

In this work, we (i) experimentally validate the ability of A. baumannii to utilize ethanolamine as a sole carbon, nitrogen, and energy source; (ii) show that the increased expression of *eutBC* is required for A. baumannii to utilize ethanolamine as a sole carbon source; (iii) show that *Ab*EAL is sufficient to support ethanolamine utilization in the absence of all other Eut proteins; (iv) determine that the A. baumannii ethanolamine ammonia-lyase *Ab*EutBC localizes to the cell membranes of A. baumannii and *S.* Typhimurium; (v) show that ethanolamine catabolism relies on the availability of an AdoCba and that the A. baumannii genome encodes the functions needed to assemble the nucleotide loop of an AdoCba from a precursor such as cobinamide; (vi) show that A. baumannii synthesizes a functional PduO-type ATP:Co(I)rrinoid adenosyltransferase; and (vii) show that *Ab*EutBC can utilize various adenosylcobamides and that A. baumannii predominantly makes cobalamin from cobinamide in the absence of exogenous base supplementation.

## RESULTS

### Chromosomal expression of *eutBC* allows A. baumannii to use ethanolamine as a sole nitrogen source but not as a carbon and energy source.

**FIG 4:**
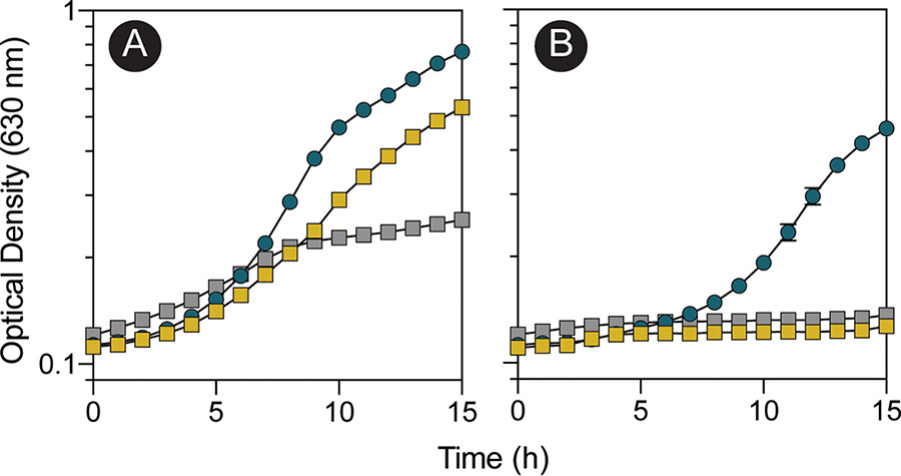
A. baumannii can use ethanolamine as a sole source of carbon or nitrogen. A. baumannii was grown on M9 minimal medium with ethanolamine as the sole source of nitrogen (A) or carbon and energy (B). Yellow squares, wild-type A. baumannii ATCC 17978; gray squares, Δ*eutBC*; blue circles, Δ*eutBC*/pAbeutBC3. For plasmid maintenance, ampicillin was added to cultures at 100 μg/mL. Experiments were performed in biological and technical triplicates. Error bars represent the standard deviations between replicates.

To determine whether A. baumannii could use EA as a nitrogen source, growth was assessed in minimal medium containing M9 salts made without NH_4_Cl but supplemented with ethanolamine as the sole source of nitrogen (Fig. 4A). To determine whether A. baumannii could use EA as a carbon and energy source, M9 minimal medium (containing NH_4_Cl) was supplemented with ethanolamine as the sole source of carbon and energy (Fig. 4B). The growth of A. baumannii with ethanolamine depended on wild-type alleles of the *eutBC* genes (Fig. 4A and B, gray squares), which encode the subunits of the adenosylcobalamin (AdoCbl)-dependent EAL enzyme subunits. Chromosomal levels of *eutBC* expression were sufficient to support growth with ethanolamine as a nitrogen source (Fig. 4A, yellow squares). When *eutBC* was overexpressed in *trans*, the cultures reached a higher density (Fig. 4A, blue circles). In contrast, A. baumannii grew with ethanolamine as the sole source of carbon and energy in medium supplemented with cyanocobalamin (CNCbl) only when *eutBC* was overexpressed (Fig. 4B, blue circles versus yellow squares).

### Spontaneous A. baumannii mutant strains grow robustly on ethanolamine when *eutBC* expression is driven by the IS*Aba1* element.

**FIG 5:**
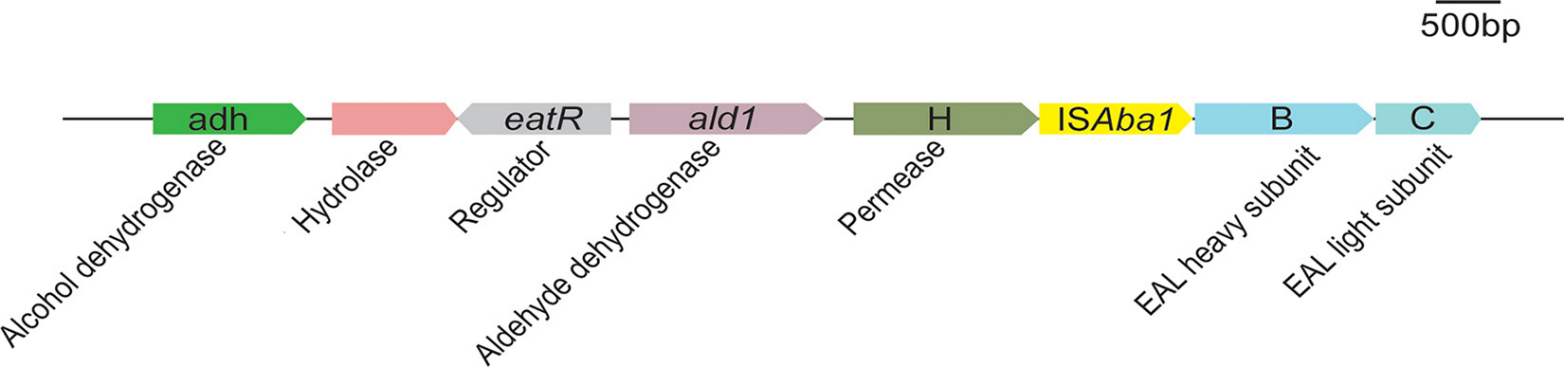
Spontaneous insertion of IS*Aba1* upstream of *eutBC* facilitates increased expression sufficient for growth using ethanolamine as a sole carbon source. Genes are presented at scale (see the scale bar).

We isolated spontaneous mutant strains of A. baumannii that abolished the extended lag time of the wild-type strain growing on ethanolamine as a carbon and energy source (Fig. 4B). The putative mutants were obtained after ~100 h of incubation in liquid medium at 37°C. To test the stability of the causative mutation(s), we passaged the mutant strain several times on solid and liquid rich medium devoid of ethanolamine. This strain maintained the ability to rapidly grow with ethanolamine as a carbon and energy source. These results suggested that the phenotype was the result of one or more stable mutations. To determine the nature of the mutation(s), we sequenced the genome of the mutant strain, and the analysis of the sequence revealed an insertion of the IS*Aba1* transposable element upstream of the *eutBC* genes, between the 3′ end of the *eutH* gene (which encodes a putative ethanolamine permease) and the start of *eutB* (which encodes the putative large subunit of ethanolamine ammonia-lyase) (Fig. 5; see also Fig. S5 in the supplemental material). This element was inserted immediately after the final nucleotide of *eutH*, leaving the 16-bp length upstream of *eutB* undisrupted.

10.1128/mbio.01793-22.5 FIG S5 IS*Aba1* insertion sequence found upstream of *eutBC* in the A. baumannii evolved strain. The inserted sequence is highlighted in pink. The final 3 nucleotides of *eutH* are highlighted in red. Predicted σ^70^ −35 (blue) and −10 (yellow) sequences are noted (22). The start codon of *eutB* is highlighted in green. Download FIG S5, PDF file, 0.3 MB. Copyright © 2022 Villa and Escalante-Semerena. 2022 Villa and Escalante-Semerena. https://creativecommons.org/licenses/by/4.0/ This content is distributed under the terms of the Creative Commons Attribution 4.0 International license.

Previous reports have shown that the IS*Aba1* element contains a σ^70^ promoter at the 3′ end of the gene and that this promoter is used to increase the expression of downstream genes (22). This element is frequently associated with the increased expression of antibiotic resistance genes in a clinical setting (22–26). The predicted σ^70^ sites found in IS*Aba1* are illustrated in Fig. S5.

**FIG 6:**
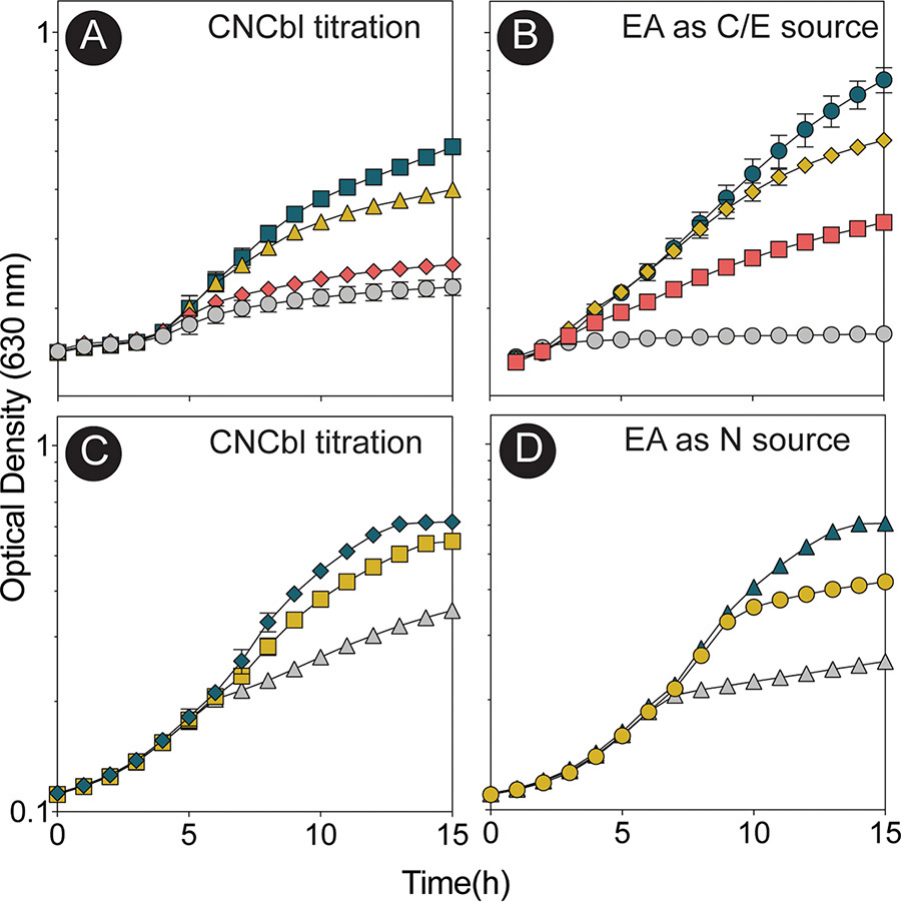
Analysis of cobamide and ethanolamine requirements. (Top) Cobalamin and EA requirements of the strain carrying the IS*Aba1* element during growth on EA as a sole carbon source. (A) CNCbl was provided at the following concentrations: none (gray circles), 0.5 nM (orange diamonds), 5 nM (mustard triangles), and 10 nM (blue squares). (B) EA was tested as a carbon and energy (C/E) source at the following concentrations: none (gray circles), 10 mM (orange squares), 25 mM (mustard diamonds), and 75 mM (blue circles). (Bottom) Cobalamin and EA requirements of the parental A. baumannii strain during growth with EA as a sole nitrogen source. (C) Titration of CNCbl at the following concentrations: none (gray triangles), 0.5 nM (mustard squares), and 5 nM (blue diamonds). (D) The following EA concentrations were tested: none (gray triangles), 1 mM (mustard circles), and 10 mM (blue triangles). In all cases, experiments were performed in biological triplicate with technical triplicates. Error bars indicate the standard deviations between replicates.

We used the strain with the IS*Aba1* element driving the synthesis of *Ab*EutBC to determine the optimal concentrations of ethanolamine as a sole carbon source and CNCbl during growth in minimal medium (Fig. 6A and B). Strong growth was observed at 10 nM CNCbl and 75 mM ethanolamine as the sole source of carbon and energy (Fig. 6A, blue squares, and Fig. 6B, blue circles). As little as 0.5 nM CNCbl and 10 mM EA promoted robust growth when EA was used as the sole source of nitrogen by the A. baumannii parental strain (Fig. 6C, yellow squares, and Fig. 6D, blue circles).

### Assessment of A. baumannii EutBC function in a heterologous host.

**FIG 7:**
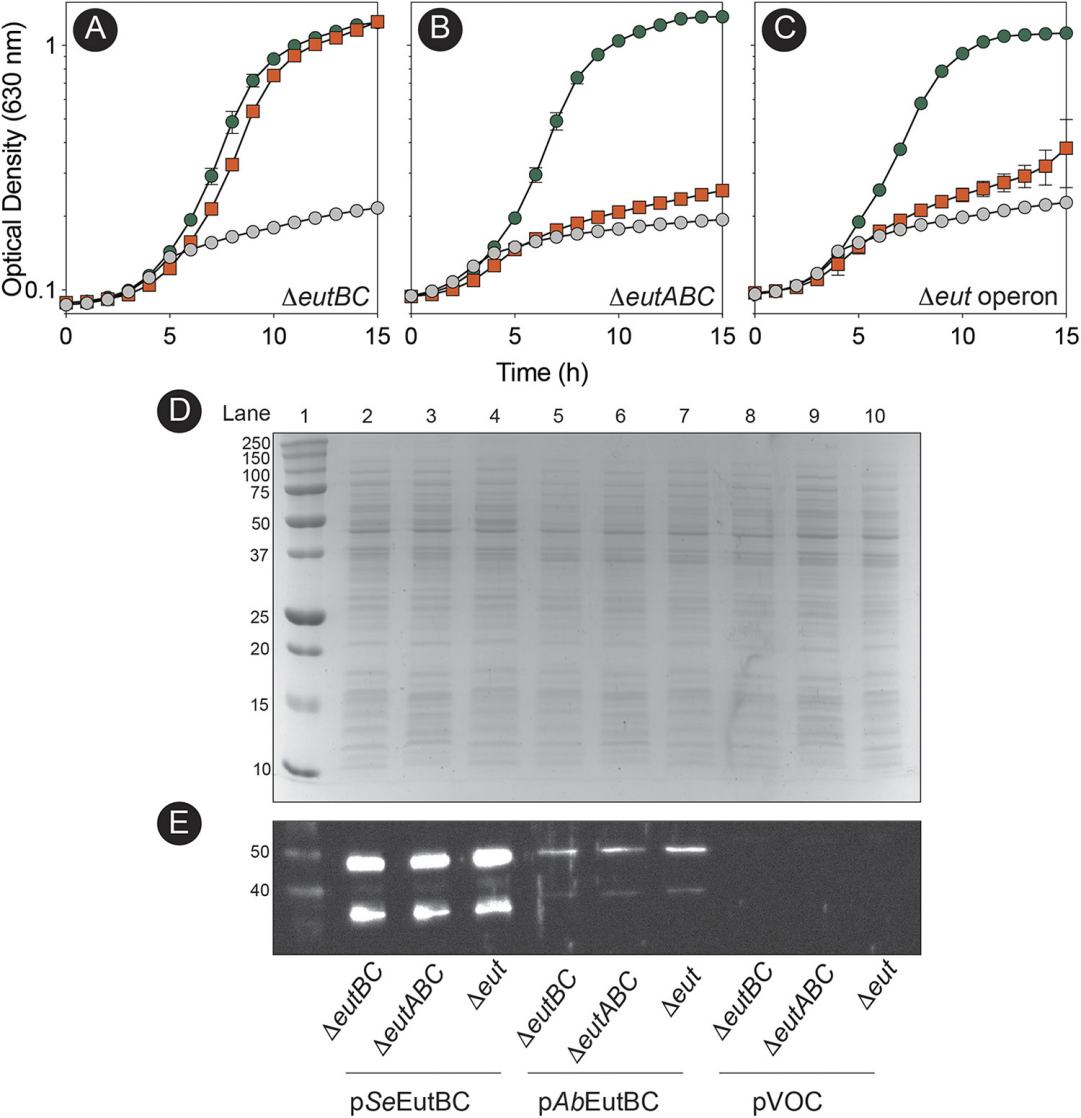
The *Ab*EutBC enzyme supports robust growth of *S.* Typhimurium *eut* mutants when ethanolamine is the sole source of nitrogen and does not require EutA reactivase function to remain active. (A to C) Strains were grown in nce minimal medium containing ethanolamine as the sole source of nitrogen. In all panels, the growth behaviors of strains carrying plasmid pEUT269 (*S*. Typhimurium *eutBC*^+^) are represented by orange squares, the strain that carried plasmid pAbEUTBC1 (A. baumannii *eutBC*^+^) is represented by green circles, and the strain that carried the empty cloning vector is represented by gray circles. The background of each strain tested is indicated at the bottom right-hand side of the panel. For plasmid maintenance, ampicillin was added to cultures at 100 μg/mL. In all cases, each strain was grown in biological and technical triplicate. Error bars indicate the standard deviations between replicates. (D) SDS-PAGE gel of crude lysates from strains used for growth analysis. Lane 1 contains molecular mass standards reported in kilodaltons. (E) Anti-EAL Western blot of crude lysates from strains used for growth analysis.

We used well-characterized *eut* mutant strains of *S.* Typhimurium to assess the level of activity of the *Ab*EutBC enzyme in different genetic backgrounds, namely, a Δ*eutBC* strain, a Δ*eutABC* strain, or a strain carrying a deletion of the entire *eut* operon (Δ*eut*) (Fig. 7). *Ab*EutBC supported the growth of an *S.* Typhimurium Δ*eutBC* strain with EA as the sole source of nitrogen as well as *Se*EutBC did (Fig. 7A, orange squares versus green circles). Notably, the *Ab*EutBC enzyme remained active in the absence of the *Se*EutA reactivase (Fig. 7B, green circles), while, as expected, the *Se*EutBC enzyme did not support growth in the absence of *Se*EutA (Fig. 7B, orange squares). Most intriguingly, *Ab*EutBC alone supported ethanolamine utilization in an *S.* Typhimurium strain in which the entire *eut* operon was deleted. Consistent with the need for *Se*EutA and other *eut* operon gene products, *Se*EutBC supported poor growth of the Δ*eut* strain (Fig. 7C, green circles versus orange squares). *Se*EutBC components (49.4 and 32.1 kDa, respectively) were detected at higher levels in the crude lysate (Fig. 7E, lanes 2 to 4) than *Ab*EutBC (50.6 and 30.1 kDa, respectively) (Fig. 7E, lanes 5 to 8). This was likely due to a stronger reaction to the anti-EAL antibody, as this antibody was generated against *Se*EutBC. The reduced signal observed for *Ab*EutC was likely due to protein instability under the tested conditions, as the theoretical pI of *Ab*EutC is 6.96. The loading buffer and gel stacking layer in this experiment were both pH 6.8.

### Genes associated with ethanolamine utilization are cotranscribed.

**FIG 8:**
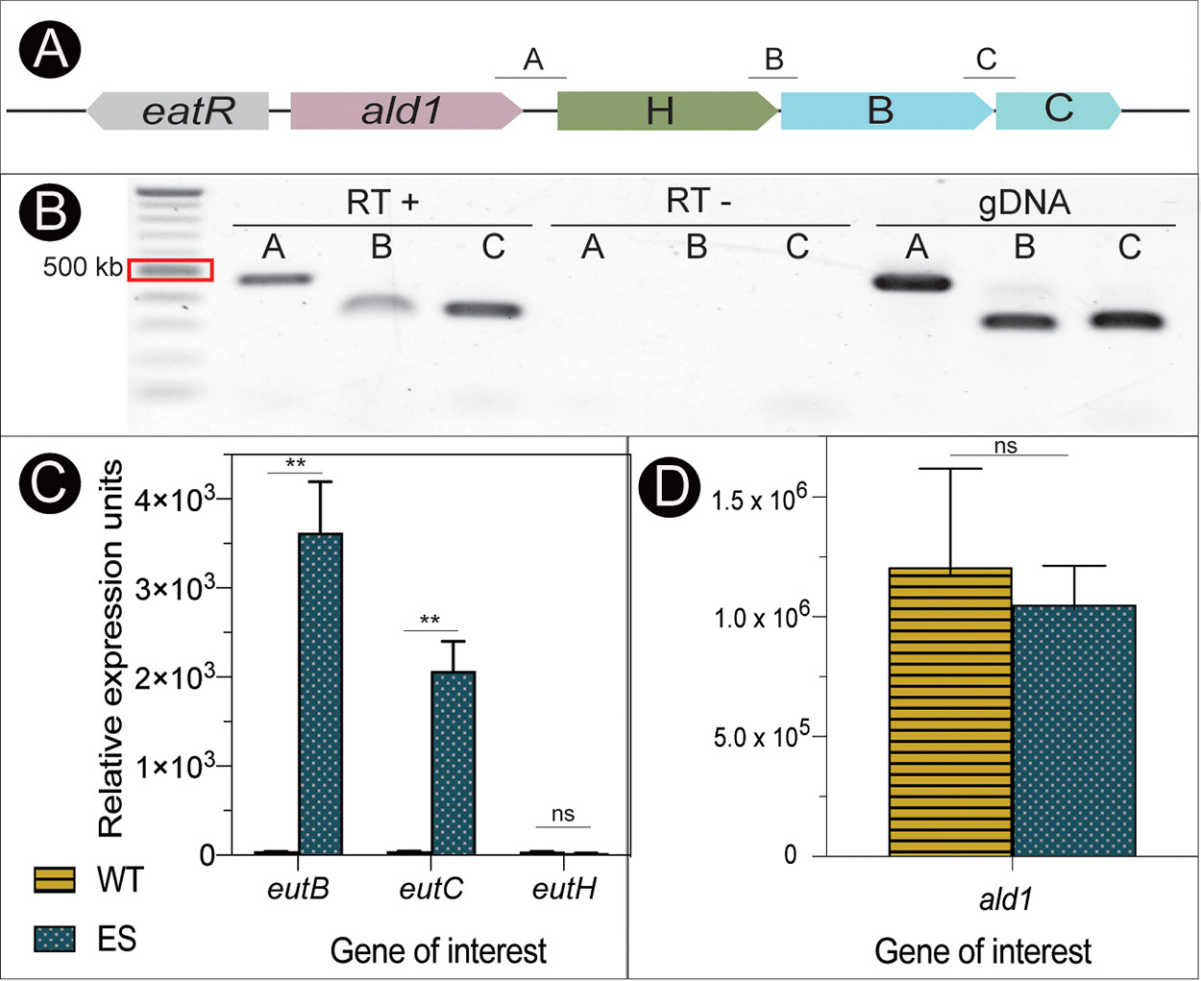
Expression of the putative A. baumannii *eut* genes. (A) Schematic of intergenic primers to test the putative *eut* operon. (B) Gel image of amplified products using intergenic primers and RNA extracted from A. baumannii incubated with (RT +) or without (RT −) reverse transcriptase or gDNA as the template. (C) RT-qPCR results of *eutB*, *eutC*, and *eutH* gene expression in wild-type (WT) A. baumannii grown on pyruvate (yellow stripes) or the evolved strain (ES) grown on ethanolamine as a sole carbon source (blue dots). (D) RT-qPCR results of *ald1* expression in wild-type A. baumannii grown on pyruvate (yellow stripes) or the evolved strain grown on ethanolamine as a sole carbon source (blue dots). **, *P* value of <0.01; ns, not significant.

To test whether the putative *eut* genes were cotranscribed, primers were generated to amplify the intergenic regions between *ald1* and *eutH*, *eutH* and *eutB*, and *eutB* and *eutC* (Fig. 8A). As previously shown in Pseudomonas aeruginosa, all four genes were transcribed as a polycistronic mRNA and expressed from a promoter upstream of *ald1* in wild-type A. baumannii (Fig. 8B) (7). We used quantitative reverse transcription PCR (RT-qPCR) to determine that the evolved strain (ES) overexpresses *eutBC* (Fig. 8C). In the absence of EA, wild-type A. baumannii produced extremely low levels of *eutBC* transcripts (Fig. 8C, yellow striped bars). The evolved strain carrying an Is*Aba1* insertion upstream of *eutBC* grown with ethanolamine as a sole carbon source showed significant increases in *eutBC* expression (100-fold and 64-fold, respectively) compared to the parental strain, while *eutH* levels were very low in both strains and under both conditions tested. We tested the expression of the upstream *ald1* gene to determine whether this might act as an acetaldehyde dehydrogenase gene and aid in the management of reactive intermediates during EA catabolism. Interestingly, *ald1* was expressed at high levels in wild-type A. baumannii even in the absence of EA (Fig. 8D); therefore, fluctuations in gene expression in response to EA may not be detectable under the conditions used. This suggested that under some conditions, *ald1* and *eutHBC* were cotranscribed, but the expression of *ald1* may be subject to other regulatory mechanisms.

### Transmission electron microscopy reveals the absence of metabolosome structures in A. baumannii grown with ethanolamine.

**FIG 9:**
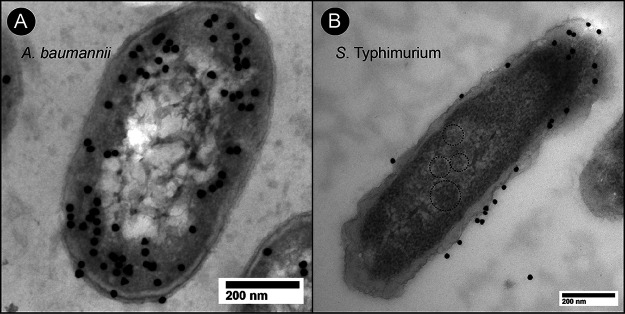
A. baumannii EutBC localizes to the cell membrane. TEM images of A. baumannii Δ*eutBC* (JE26441) (A) and *S.* Typhimurium Δ*eutBC* (JE26302) (B) grown on EA and expressing *Ab*EutBC in *trans* are shown. For plasmid maintenance, kanamycin (50 μg/mL) was added to A. baumannii cultures, and ampicillin (100 μg/mL) was added to *S.* Typhimurium cultures. Grids were labeled with a primary antibody generated against *S.* Typhimurium EutBC and a gold-conjugated secondary antibody and poststained with uranyl acetate. Metabolosomes are indicated with dotted circles.

We used transmission electron microscopy (TEM) and immunogold labeling to localize the *Ab*EutBC proteins within the A. baumannii cell. Previous reports have shown that *Se*EutBC was associated with the metabolosome (27), which can be visualized by TEM (6). No similar structures were visible in A. baumannii cells in the presence of EA as a sole carbon source (Fig. 9A; Fig. S6A and B). In addition, *Ab*EutBC localized primarily to the periphery of cells, with some protein apparently being interspersed throughout the cytosol. We further investigated localization within the cell by overexpressing *Ab*EutBC in an *S.* Typhimurium Δ*eutBC* strain (Fig. 9B; Fig. S6C and D). It is important to note that the genome of this strain of *S.* Typhimurium harbors the genes for the metabolosome shell. When expressed in the presence of the *eut* metabolosome, *Ab*EutBC did not associate with the metabolosome structure; strikingly, it was associated exclusively with the cell membrane (Fig. 9B). No EAL was detected in control samples treated with a secondary antibody only (Fig. S7).

10.1128/mbio.01793-22.6 FIG S6 A. baumannii EutBC localizes to the cell membrane. TEM images of A. baumannii (A and B) and *S.* Typhimurium (C and D) expressing *Ab*EutBC in *trans* are shown. Grids were labeled with a primary antibody generated against *S.* Typhimurium EutBC and a gold-conjugated secondary antibody. For plasmid maintenance, kanamycin (50 μg/mL) was added to A. baumannii cultures, and ampicillin (100 μg/mL) was added to *S.* Typhimurium cultures. Download FIG S6, JPG file, 0.5 MB. Copyright © 2022 Villa and Escalante-Semerena. 2022 Villa and Escalante-Semerena. https://creativecommons.org/licenses/by/4.0/ This content is distributed under the terms of the Creative Commons Attribution 4.0 International license.

10.1128/mbio.01793-22.7 FIG S7 TEM nonspecific controls. TEM images of A. baumannii Δ*eutBC* (JE26441) (top) and *S.* Typhimurium Δ*eutBC* (JE26302) (bottom) expressing *Ab*EutBC in *trans* grown in minimal medium containing ethanolamine are shown. Cells were processed as described in Materials and Methods and were treated with goat anti-rabbit IgG-gold secondary antibody in the absence of anti-EutBC primary antibody. Download FIG S7, PDF file, 1.0 MB. Copyright © 2022 Villa and Escalante-Semerena. 2022 Villa and Escalante-Semerena. https://creativecommons.org/licenses/by/4.0/ This content is distributed under the terms of the Creative Commons Attribution 4.0 International license.

### A. baumannii synthesizes cobamides from the precursor cobinamide.

The *Se*EutBC enzyme requires AdoCbl as its cofactor (28). Our bioinformatics analysis of the A. baumannii genome indicated that this bacterium lacked the functions needed for the *de novo* synthesis of the corrin ring of cobamides but appeared to encode the function needed to adenosylate the corrin ring, i.e., to attach the adenosyl (Ado) moiety from ATP to the Co ion of the ring, and the functions required for the assembly of the nucleotide loop (29).

**FIG 10:**
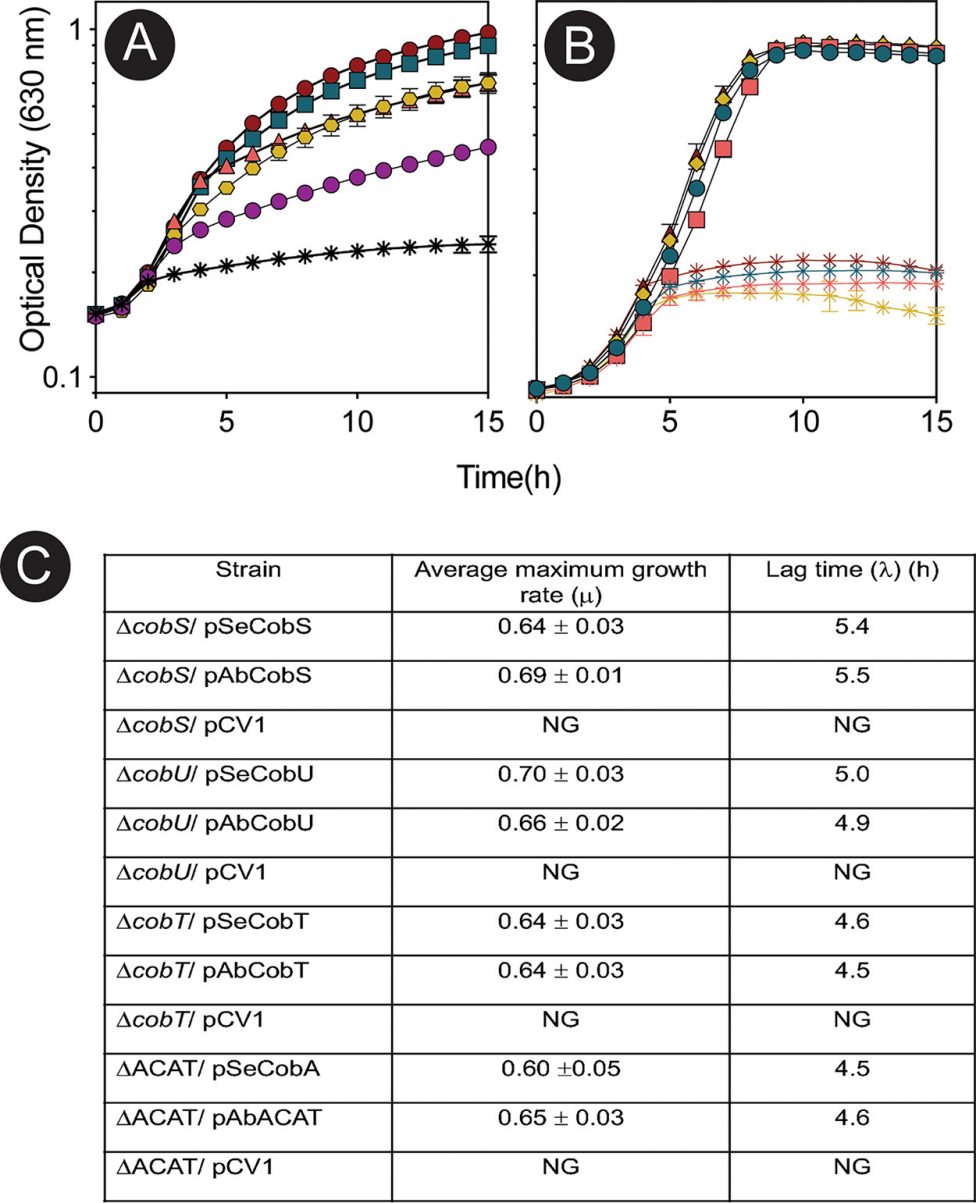
A. baumannii can salvage cobinamide to form a complete cobamide. (A) EA utilization by the A. baumannii evolved strain (ES) grown in minimal medium containing EA as the sole source of carbon and energy. To test the utilization of various bases, cultures were supplemented with cobalamin (red circles) or cobinamide with the addition of 5-methoxybenzimidazole (cyan squares), adenine (green triangles), 5,6-dimethylbenzimidazole (yellow hexagons), no base (purple circles), or no cobamide (gray diamonds). (B) Complementation of A. baumannii NLA genes in *S.* Typhimurium. Red squares, Δ*cobS*/pAbCobS; open inverted orange triangles, Δ*cobS*/vector; cyan squares, Δ*cobU*/pAbCobU; black diamonds, Δ*cobU*/vector; green triangles, Δ*cobT*/pAbCobT; blue hexagons, Δ*cobT*/vector; yellow diamonds, ΔACAT/pAbACAT; purple circles, ΔACAT/vector. Ampicillin (100 μg/mL) was added to cultures for plasmid maintenance. For each strain, the growth behavior of three separate biological replicates in technical triplicate was analyzed. Error bars represent the standard deviation between replicates. (C) Growth behavior of *S.* Typhimurium strains expressing A. baumannii genes. Growth rates were determined using online tools (https://scott-h-saunders.shinyapps.io/gompertz_fitting_0v2/).

The formation of a complete coenzyme from the precursor cobinamide requires the nucleotide loop assembly (NLA) pathway (Fig. S4). Under the conditions tested, A. baumannii salvaged the precursor cobinamide and other nitrogenous bases to form a functional coenzyme and facilitate growth with EA, demonstrating that NLA pathway enzymes were functional (Fig. 10A). We also tested the activity of NLA enzymes using heterologous expression in a MetH-dependent *S.* Typhimurium strain. The A. baumannii NLA enzymes CobU, CobS, and CobT compensated for the absence of the *cobU*, *cobS*, and *cobT* genes of *S.* Typhimurium (Fig. 10B and C). Furthermore, the A. baumannii *pduO-*type ACAT supported EA metabolism in an *S.* Typhimurium ACAT-null strain. The NLA enzymes CobU and CobS require adenosylated substrates (30, 31); thus, salvaging of the precursor cobinamide depended on the function of the A. baumannii ACAT under these conditions (Fig. 10B and C). These results demonstrated that A. baumannii NLA and ACAT homologues were functional.

### Cobalamin is produced by A. baumannii in the absence of nucleobase supplementation.

Prokaryotes can utilize numerous nucleobases to yield a functional coenzyme (32). As shown in Fig. 10A, various bases can be used by A. baumannii to form a functional coenzyme. Salvaging was performed in the absence of exogenous nucleobases; however, this led to diminished growth (Fig. 10A, purple circles). To determine the identity of this cobamide produced by A. baumannii in the absence of exogenous nucleobases, we extracted cobamides and separated them using reverse-phase high-performance liquid chromatography (RP-HPLC) as described in Materials and Methods. A compound eluted off the column 33 min after sample injection. The retention time was equal to the retention time of commercially available CNCbl (Fig. S8A and B). This peak was collected, cleaned, and analyzed using matrix-assisted laser desorption ionization–time of flight (MALDI-TOF) mass spectrometry, confirming that cobalamin, the cobamide containing 5,6-dimethylbenzimidazole (DMB) as its nucleobase, was the cobamide synthesized by A. baumannii in the absence of exogenous nucleobases (Fig. S8C).

10.1128/mbio.01793-22.8 FIG S8 (A) RP-HPLC chromatogram of the pure standards CN_2_Cbi (blue) and CNCbl (red). (B) RP-HPLC chromatogram of extracts of strain JE26013 (A. baumannii) grown with ethanolamine as a sole carbon source and CN_2_Cbi. (C) MALDI-TOF mass spectrum of the peak isolated at 33 min. Download FIG S8, PDF file, 0.1 MB. Copyright © 2022 Villa and Escalante-Semerena. 2022 Villa and Escalante-Semerena. https://creativecommons.org/licenses/by/4.0/ This content is distributed under the terms of the Creative Commons Attribution 4.0 International license.

## DISCUSSION

### A. baumannii catabolizes ethanolamine as a source of carbon and nitrogen.

Based on the data presented here, we surmised that the wild-type strain of A. baumannii used in this work synthesizes insufficient *Ab*EutBC to support growth with EA as the sole carbon and energy source. It is possible, however, that in its natural environment, A. baumannii uses EA mainly as a source of nitrogen, and for that purpose, enough EAL is produced from chromosomal expression levels. Our data show that A. baumannii can use the IS*Aba1* element to increase the expression of *eutBC* if selective pressure for the use of ethanolamine as a carbon and energy source arises in its environment. Previous studies have identified IS*Aba1* upstream of antibiotic resistance genes in patient samples, suggesting that this is a clinically relevant adaptive mechanism used by this organism during human infection (23). The insertion of an IS*Aba1* element does not affect the expression of the upstream *eutH* and *ald1* genes. The low level of *eutH*, even in the presence of EA, suggests that, under the conditions tested, active transport is not required and that sufficient EA diffuses across the membrane into the cell. We have shown that the requirements for both cobamide and EA concentrations are lower in A. baumannii than in *S.* Typhimurium. This reduced need may reflect the faster access of enzymes to these molecules due to the lack of restriction by the metabolosome.

### *Ab*EAL is sufficient to support ethanolamine metabolism in the absence of a metabolosome or EAL reactivase.

We did not identify genes encoding homologues of metabolosome shell proteins in the A. baumannii genome, suggesting to us that A. baumannii may be able to perform this metabolism without containment in a microcompartment. The N terminus of *Se*EutC contains a signal sequence involved in localization to the microcompartment (33). Consistent with the lack of any genes encoding microcompartment shell proteins, the *Ab*EutC sequence also lacks any predicted N-terminal signal sequence (see Fig. S1D in the supplemental material). We showed here that *Ab*EAL does not require a reactivase; furthermore, *Ab*EAL alone can support EA utilization in the absence of the entire *S.* Typhimurium *eut* operon. This suggests that either the *Ab*EAL enzyme directly quenches the acetaldehyde generated from EA or A. baumannii reduces cellular damage caused by acetaldehyde using an alternative mechanism. The mechanism by which *Ab*EAL functions in the absence of a physical metabolosome is intriguing. Using TEM, we showed that A. baumannii does not form metabolosomes and that the enzyme does not associate with metabolosomes when expressed in *S.* Typhimurium (Fig. 9). Based on these data, we surmise that the *Ab*EAL enzyme acts independently of other Eut components in *S.* Typhimurium. The membrane association of *Ab*EAL was more pronounced when expressed in *S.* Typhimurium. It is possible that this is due to the association of *Ab*EAL with other B_12_-related enzymes not found in A. baumannii. We hypothesize that in A. baumannii, other enzymes, such as the cotranscribed aldehyde dehydrogenase, associate with *Ab*EAL to facilitate the swift quenching of reactive molecules and prevent cellular damage. To our knowledge, this is the first report of ethanolamine metabolism occurring in an organism that does not form a metabolosome.

### Multiple cobamides can be formed by the A. baumannii salvaging pathway and used as a coenzyme by *Ab*EAL.

A. baumannii carries the genes for nucleotide loop assembly as well as a functional PduO-type ACAT. Due to the apparent absence of other genes related to propanediol metabolism, we instead refer to this protein as a general ACAT (AcaT). The unique organization of these genes in A. baumannii is notable, as the *cobUST* genes are typically organized immediately adjacent to each other. We have shown experimental evidence that the *cobU*, *cobT*, and *cobS* gene products facilitate the use of various nucleobases to form the nucleotide loop and that the resulting cobamides function as coenzymes for *Ab*EAL. This is consistent with a previous report in which *Se*EAL was shown to also use pseudocobalamin (34). Structural studies would provide insight into the ability of this enzyme complex to utilize multiple cobamide coenzymes.

### Ethanolamine metabolism and pathogenesis.

Understanding the metabolic capabilities of A. baumannii is of great interest as we attempt to combat antimicrobial resistance. EA has been shown to play a role in the environmental sensing and pathogenesis of *S.* Typhimurium and Escherichia coli (3, 35, 36). Although A. baumannii is not generally associated with gastrointestinal disease or part of a normal gut microbiome, it has been found to inhabit the digestive tract of long-term-ICU (intensive care unit) patients (37), indicating that the intestine may be a reservoir for this nosocomial pathogen. EA breakdown may be an important metabolic capability for A. baumannii during the course of colonization or infection, and if so, the enzymes studied here could serve as effective therapeutic targets.

## MATERIALS AND METHODS

### Bacterial strains and growth conditions.

The *S.* Typhimurium and A. baumannii strains used in this study are listed in Table S1 in the supplemental material. All A. baumannii strains used were derivatives of A. baumannii ATCC 17978. All *S.* Typhimurium strains used were derivatives of S. enterica subsp. *enterica* serovar Typhimurium strain LT2. E. coli DH5α was used for plasmid construction. All cultures were grown at 37°C. A. baumannii was grown in lysogeny broth (LB) (38, 39) or M9 minimal medium (40). To test EA as a carbon source, M9 minimal medium was supplemented with MgSO_4_ (1 mM), CaCl_2_ (10 μM), EA-HCl (90 mM, unless otherwise noted), LB (1%, vol/vol), and cyanocobalamin (CNCbl) (100 nM, unless otherwise noted). To test EA as a nitrogen source, A. baumannii was grown in nitrogen-free M9 salts supplemented with MgSO_4_ (1 mM), CaCl_2_ (10 μM), sodium pyruvate (20 mM), CNCbl (10 nM, unless otherwise noted), and EA-HCl (30 mM, unless otherwise noted). *S.* Typhimurium strains were grown on nutrient broth (NB; Difco) or no-carbon essential (NCE) minimal medium (41); E. coli was grown in LB. When antibiotics were used, their concentrations were indicated for each experiment. Unless otherwise noted, isopropyl β-d-1-thiogalactopyranoside (IPTG) was used at 1 mM, and l-(+)-arabinose was used as an inducer of gene expression at 0.5 mM. Unless otherwise specified, all chemicals were purchased from Sigma-Aldrich Chemical Company.

10.1128/mbio.01793-22.9 TABLE S1 Strains and plasmids used in this study. ^a^All strains and plasmids were constructed during the course of this work unless otherwise stated. *Se*, Salmonella enterica subsp. *enterica* serovar Typhimurium strain LT2 or its derivatives (which are also abbreviated as *S.* Typhimurium); *Ab*, Acinetobacter baumannii strain ATCC 17978 or its derivatives; IS*Aba1*, insertion sequence Acinetobacter baumannii a1. Download Table S1, PDF file, 0.08 MB. Copyright © 2022 Villa and Escalante-Semerena. 2022 Villa and Escalante-Semerena. https://creativecommons.org/licenses/by/4.0/ This content is distributed under the terms of the Creative Commons Attribution 4.0 International license.

### Construction of A. baumannii deletion strains.

Deletions were constructed as described previously (42, 43). A. baumannii harboring plasmid pRecABtet was transformed with a fragment containing flanking regions of the gene of interest fused to the kanamycin (Km) resistance gene from plasmid pKD4 by electroporation. Cells were recovered using superoptimal broth with catabolite (SOC) repression medium (tryptone [20 g/L, wt/vol], yeast extract [5 g/L], NaCl [0.5 g/L], glucose [20 mM]) at 37°C with shaking at 220 rpm for 2 to 4 h. Following incubation, cells were plated onto LB (Difco) agar plates containing Km (25 μg/mL) and IPTG (2 mM). Kanamycin-resistant (Km^r^) transformants were patched onto plates containing tetracycline (Tc) (10 μg/mL), and tetracycline-sensitive (Tc^s^) Km^r^ colonies were analyzed further. To resolve the kanamycin cassette, plasmid pFLPtet was moved into the strains by electroporation. Cells were recovered with SOC medium (2 mL) for 2 to 4 h at 37°C with shaking at 220 rpm and plated onto LB agar containing tetracycline (10 μg/mL). Cells were streaked for isolation onto plates containing tetracycline (10 μg/mL) and IPTG (2 mM) to induce the expression of *flp*. Isolated colonies were streaked onto LB and then patched onto LB agar containing kanamycin (30 μg/mL) or tetracycline (10 μg/mL). We isolated genomic DNA (gDNA) from Tc^s^ Km^s^ colonies as described previously (42), followed by PCR amplification and DNA sequencing to verify the disruption of the gene of interest and the loss of the Km resistance cassette.

### Plasmid construction.

Plasmids used in this study are listed in Table S1. Primers used for the construction of strains, the construction of complementation plasmids, and PCR analysis are listed in Table S2. To test complementation in *S.* Typhimurium, each gene of interest from A. baumannii was cloned into cloning vector pCV1 (44) using BspQI restriction sites and electroporated into E. coli DH5α. Plasmids were miniprepped using a Wizard Plus SV Minipreps DNA purification system (VWR) and sequenced to verify each insert. Positive plasmids were electroporated into an *S.* Typhimurium strain carrying a deletion of the homologous gene. Cells containing the plasmid were selected for by the addition of ampicillin (100 μg/mL) to the growth medium.

10.1128/mbio.01793-22.10 TABLE S2 Primers used in this study. ^a^All primers were synthesized by Integrated DNA Technologies (Coralville, IA). Download Table S2, PDF file, 0.03 MB. Copyright © 2022 Villa and Escalante-Semerena. 2022 Villa and Escalante-Semerena. https://creativecommons.org/licenses/by/4.0/ This content is distributed under the terms of the Creative Commons Attribution 4.0 International license.

Complementation studies in A. baumannii were performed as described previously (45). A. baumannii *eutBC* was inserted into pMMB67EH using EcoRI and SalI (Thermo Fisher) restriction digests, and ligation was then performed using T4 DNA ligase (Thermo Fisher) according to the manufacturer’s instructions. Chemically competent E. coli DH5α was transformed and plated onto Tc (10 μg/mL). Selected colonies were miniprepped and sequenced to verify the insertion. Verified plasmids were used to transform electrocompetent A. baumannii Δ*eutBC* as described above, and cells were recovered in SOC medium for 2 to 4 h and plated onto LB agar containing tetracycline.

### Analysis of EA catabolism in A. baumannii.

A. baumannii strains were grown in LB overnight and subcultured (5%, vol/vol) into M9 minimal medium or nitrogen-free M9 minimal medium. Growth analyses were performed using a 96-well microtiter dish (Falcon) with 200 μL per well, cultures were incubated at 37°C with shaking, and increases in cell density were monitored at 630 nm for 24 h. Each growth analysis consisted of three biological replicates in technical triplicate. Concentrations of CNCbl or ethanolamine were changed where indicated to assess the minimal requirements of A. baumannii for these nutrients.

### Complementation studies in *S.* Typhimurium.

The heterologous expression of A. baumannii genes was performed in *S.* Typhimurium to test EAL, NLA, and ACAT functions. NB was inoculated with a single colony, and the colony was grown overnight at 37°C. The following day, 1% (vol/vol) of overnight culture (~16 h old) was transferred into minimal medium in a 96-well microtiter plate. *S.* Typhimurium growth experiments were performed in NCE minimal medium (41) containing Wolfe’s trace minerals (46), MgSO_4_ (1 mM), glycerol (22 mM), and ampicillin (100 μg/mL). Cobamide precursor salvaging was performed by assessing growth with the addition of dicyanocobinamide [(CN)_2_Cbi] and 5,6-dimethylbenzimidazole (DMB) (150 μM). Gene expression was induced with l-(+)-arabinose (0.5 μM). The growth of cultures in microtiter plates was monitored every hour at 630 nm during incubation with shaking at 37°C in a BioTek Elx808 microplate reader. To test the functionality of the A. baumannii *eutBC* gene products, minimal medium containing no-carbon nonessential NCE medium (47) with ethanolamine (30 mM) provided as the sole source of nitrogen was used. In each case, growth analysis was performed in a BioTek Elx808 microplate reader with three biological replicates in technical triplicate.

### Sequencing of A. baumannii genomes.

Strains of A. baumannii able to grow on ethanolamine as the sole source of carbon and energy were grown overnight in LB (10 mL) at 37°C with shaking at 180 rpm. Cells were harvested by centrifugation at 3,000 × *g* using a tabletop Eppendorf 5810R centrifuge for 30 min at room temperature. Genomic DNA was isolated using a blood and cell culture DNA midi kit and a genomic DNA buffer set (Qiagen) according to the manufacturer’s protocol for the preparation of Gram-negative samples. The quality of genomic DNA was measured using an Invitrogen Qubit model 4 fluorometer, and only samples containing >10% (wt/vol) double-stranded DNA were used for whole-genome sequencing (WGS). DNA-end repair was performed using NEBNext FFPE (formalin-fixed, paraffin-embedded) DNA repair mix and NEBNext Ultra II end repair (New England BioLabs [NEB]) according to the manufacturer’s instructions. The repair reaction was allowed to proceed for 30 min at 20°C and 30 min at 65°C. DNA was isolated by mixing with AMPure XP beads, washing with 70% (vol/vol) ethanol, and eluting off beads with Ambion nuclease-free water. The eluate containing end-repaired DNA was quantified using a Qubit fluorometer. Barcodes were attached using NEB blunt/TA ligase and master mix (New England BioLabs) according to the manufacturer’s instructions, and the mixture was incubated at 25°C for 15 min. DNA was purified from the reaction mix using AMPure beads according to the manufacturer’s instructions and eluted in 25 μL nuclease-free water. Barcoded DNA was quantified using a Qubit fluorometer. Equimolar amounts of barcoded samples were pooled to a total of 1,050 ng in 65 μL. Adapters were attached to barcoded DNA using adapter mix, NEBNext ligation buffer, and NEBNext quick T4 DNA ligase (New England BioLabs), and the mixture was incubated at 20°C for 15 min. Following incubation, DNA was isolated from the reaction mixture using AMPure XP beads as described above. Beads were washed with long-fragment buffer (Nanopore) twice before elution with elution buffer (Nanopore). The final concentration of the library was quantified using a Qubit fluorometer. Libraries were loaded into an R10.3 flow cell on a MinION Mk1C system (Nanopore Technologies).

### Antibody preparation.

Purified *S.* Typhimurium EAL was provided to Envigo for the production of rabbit polyclonal antibodies. Antiserum was precleared against JE8094 (Δ*eutBC*). Cells were grown at 37°C with shaking at 180 rpm in minimal NCE medium containing ribose (20 mM), EA (30 mM), and CNCbl (200 nM) to an optical density at 600 nm (OD_600_) of ~1.0. Cells were incubated on ice for 15 min and then harvested by centrifugation at 2,000 × *g* for 15 min in a 5810 R centrifuge (Eppendorf). Cells were resuspended in 100 μL Tris-HCl buffer (50 mM [pH 7.5] at 4°C) containing SDS (2%, wt/vol). To lyse cells, the suspension was boiled for 5 min and then incubated on ice for 10 min. The solution was brought to a final volume of 1 mL with Tris-HCl buffer (50 mM [pH 7.5] at 4°C). Antiserum was added at a 1:100 dilution, and the mixture was incubated at 4°C for 36 h. Precleared antibody was obtained by centrifugation at 16,000 × *g* for 10 min and removal of the supernatant. Precleared antibody was flash-frozen in liquid N_2_ and stored at −80°C until use.

### Western blotting.

*S.* Typhimurium strains were grown in NCE or no-carbon or no-nitrogen (NCN) medium containing EA (30 mM) as the sole source of nitrogen. Each medium was supplemented with Wolfe’s trace minerals, MgSO_4_ (1 mM), glycerol (22 mM), CNCbl (150 nM), ampicillin (100 μg/mL), and l-(+)-arabinose (0.5 μM). Cultures were inoculated with LB (2%, wt/vol) and grown overnight with shaking at 180 rpm at 37°C to an OD_600_ of 0.45 to 0.65. Cells were harvested using a 5810 R centrifuge (Eppendorf) at 2,220 × *g* for 15 min and stored at −80°C. Cells were resuspended in 1 mL sterile phosphate-buffered saline (PBS) (10 mM sodium phosphate [pH 7.4], 0.9% [wt/vol] NaCl) and centrifuged at 4,000 × *g* for 10 min in a 5415 D centrifuge (Eppendorf) at 4°C. Cell pellets were resuspended in bacterial protein extraction reagent (B-PER) (300 μL; Thermo Fisher) and vortexed for 1 min to lyse cells. The protein concentration in the crude lysate was quantified using Bradford protein assay dye (Bio-Rad Laboratories) according to the manufacturer’s instructions. Bovine gamma globulin (Thermo Fisher) was used to generate a standard curve. Cell lysates were diluted to 500 ng/μL in PBS and incubated with loading buffer (Tris [pH 6.8] [300 mM], glycerol [60%, vol/vol], EDTA [12 mM], 2-mercaptoethanol [0.85 M], and bromophenol blue [0.05%, wt/vol]) for 5 min at 95°C. The crude lysate was loaded in duplicate onto a 15% (wt/vol) SDS-PAGE gel (48). Gels were loaded with Precision-Plus protein standards (Bio-Rad Laboratories) or a SuperSignal molecular weight protein ladder (Thermo Fisher). Gels intended for Coomassie staining were loaded with 5 μg of the crude lysate; gels intended for Western blotting were loaded with 5 μg for strains complemented with *Ab*EutBC or vector-only control (VOC) and 1.25 μg for strains complemented with *Se*EutBC. A constant voltage of 200 V was applied to gels for approximately 35 min. Gels loaded with Precision-Plus protein standards were stained with brilliant blue R dye to visualize proteins. Gels loaded with the SuperSignal molecular weight protein ladder were transferred to a polyvinylidene difluoride (PVDF) membrane using a Trans-Blot Turbo system (Bio-Rad Laboratories). Following transfer, the membrane was incubated in blocking buffer composed of 0.5% (wt/vol) nonfat milk in PBST (phosphate-buffered saline [pH 7.4] containing 0.1% [vol/vol] Tween 20 [Millipore Sigma]) for 1 h at room temperature on an advanced digital shaker (VWR). The primary antibody generated against *S.* Typhimurium EAL was diluted 1:2,000 in blocking buffer, and the membrane was incubated in this solution at 4°C overnight on a tabletop shaker. The following morning, the membrane was washed three times with PBST. The membrane was incubated in PBST containing a goat anti-rabbit IgG–horseradish peroxidase conjugate (1:20,000; Invitrogen) for 1 h at room temperature on a tabletop shaker. The membrane was washed three times with PBST, incubated with SuperSignal West Pico Plus chemiluminescent substrate (Thermo Fisher) for 10 min at room temperature, and imaged using a UVP ChemStudio system (Analytik Jena) with an exposure time of 5 s.

### RNA isolation.

RNA was isolated as described previously (49). A culture grown overnight in LB medium inoculated with a single colony was subcultured (2.5%, vol/vol) in 10 mL minimal medium. Cultures grown for RT-qPCR analyses were grown in M9 minimal medium containing either ethanolamine (90 mM) or pyruvate (20 mM) as a sole carbon and energy source. For cultures used in experiments aimed at determining whether the A. baumannii putative *eut* genes were cotranscribed, wild-type A. baumannii (JE25551) was grown in no-nitrogen M9 minimal medium supplemented with 30 mM ethanolamine and 20 mM pyruvate. In all cases, cultures were incubated with shaking (200 rpm) at 37°C to an OD_600_ of 0.4 with 0.5 and harvested using a 5810 R centrifuge (Eppendorf) at 3,220 × *g* for 10 min. The supernatant was removed, and cells were stored at −80°C until they were processed. Cell pellets were lysed by resuspending the cells in boil solution (EDTA [18 mM], SDS [0.025%, vol/vol], 2-mercaptoethanol [1%, vol/vol], formamide [95%, vol/vol], RNase-free water brought to a final volume of 1 ml and heating the suspension at 95°C for 7 min. The cell suspension was centrifuged at 16,000 × *g* for 5 min at room temperature, and 100 μL of the supernatant was removed into a new tube. RNase-free water (400 μL) was added to dilute the culture, and sodium acetate was added to a final concentration of 0.3 M. Ice-cold ethanol (100%) was added (1,650 μL), and samples were vortexed briefly to mix and then incubated at −80°C overnight. Samples were centrifuged for 30 min at 16,000 × *g* at 4°C, and the supernatant was discarded. The remaining pellet was washed with 300 μL of ice-cold 70% ethanol and centrifuged at 8,000 × *g* for 5 min at 4°C. The supernatant was discarded, and the pellet was allowed to air dry. RNA pellets were resuspended in 100 μL RNase-free water and centrifuged for 1 min at 16,000 × *g* to remove any insoluble contaminants, and 90 μL was transferred into a new tube. A DNA digest was performed using the rigorous DNase treatment for Turbo DNase according to the manufacturer’s instructions (Thermo Fisher). Following DNase treatment and inactivation, samples were centrifuged at 10,000 × *g* for 1.5 min, and 90 μL was transferred into a new tube. A second sodium acetate-ethanol precipitation step was performed as described above. RNA yield and quality were analyzed using a Qubit 4 fluorometer using the RNA broad range (BR) assay kit and the RNA IQ assay kit, respectively (Invitrogen). Samples with an RNA IQ value of 6 or higher were used for RT-qPCR experiments.

### cDNA synthesis and RT-qPCR.

RNA to be used as the cDNA template was diluted to a final concentration of 12.5 ng/μL, and cDNA was synthesized using an iScript cDNA synthesis kit (Bio-Rad) according to the manufacturer’s protocol. cDNA was diluted to 2.5 ng/μL when used for RT-qPCR experiments. RT-qPCRs were performed in a 96-well plate. In each well, 20 μL of reaction mix containing 10 mL Fast SYBR green master mix (Life Technologies), gene-specific primers (500 nM), and cDNA (10 ng) was added. Gene-specific primers were designed using Primer3 software. The A. baumannii 16S rRNA gene was used as an internal control. RT-qPCRs were run using a 7500 Fast real-time PCR system (Applied Biosystems). Three biological replicates each in technical triplicate were analyzed for each strain. The cycle threshold (*C_T_*) was normalized against 16S values, and these values were transformed using the formula 2(*e* − Δ*C_T_*)/10^−6^ and reported as arbitrary expression units (EU). The mean EU from each biological replicate was used to calculate the standard error in Prism software v9 (GraphPad). Statistical significance was determined using Welch’s *t* test in Prism software v9 (GraphPad).

### Operon PCR.

Intergenic primers were generated to amplify the regions between genes within the putative operon. Five microliters of the iScript reaction product with reverse transcriptase (+RT) or without reverse transcriptase (−RT) was added to each reaction mixture. gDNA was diluted to 12.5 ng/μL, and 5 μL was added to each control reaction mixture. PCR was performed using GoTaq green master mix (Promega) according to the manufacturer’s instructions. Products were analyzed on a 1% (wt/vol) agarose DNA gel.

### Cell preparation for transmission electron microscopy.

A. baumannii was grown in M9 minimal medium containing EA as a sole carbon source. *S.* Typhimurium was grown in NCN medium supplemented with glycerol (22 mM) and EA (30 mM). In both cases, cultures were harvested at an OD_600_ of ~0.6 in a 5810 R centrifuge (Eppendorf) at 3,220 × *g* for 10 min. Cell pellets were resuspended in Sorensen’s phosphate buffer (0.1 M sodium phosphate [pH 7.4]) containing 4% paraformaldehyde and 0.2% glutaraldehyde and incubated at room temperature for 15 min. Fixed cells were centrifuged at 6,000 × *g* for 2 min. The supernatant was removed, and cells were washed twice in Sorensen’s phosphate buffer. Cells were enrobed in melted, 60°C Noble agar (4% in Sorensen’s buffer [pH 7.4]) and cut into 2-mm blocks for dehydration.

Samples were dehydrated by treatment with the following steps: 50% ethanol for 15 min, 70% ethanol for 15 min, and 80% ethanol for 10 min. The sample at each step was incubated on a slow rotator set at a 45° angle at room temperature. Cells were incubated with a mixture of LR white resin (London Resin Company, Ltd.) and 80% ethanol (2:1, vol/vol) for 1 h at room temperature. Cells were then incubated with LR white resin (100%) three times for 1 h each. Each incubation was performed at room temperature on a slow rotator set at a 45° angle. Cells were embedded in LR white resin in gelatin capsules and incubated at 50°C for 14 to 16 h. Sections of 30 to 50 nm were cut using a DiaTOME Ultra diamond knife mounted on an RMC-MT-X microtome. Sections were placed onto gold grids coated with Formvar and carbon until immunogold labeling was performed.

Grids were incubated top-down on a 25-μL drop of blocking buffer containing 20 mM Tris (pH 7.4), 150 mM NaCl, and 1% (wt/vol) bovine serum albumin (BSA) for 10 min and then washed by repeated dipping in a beaker containing 10 mL blocking buffer for 1 min. Grids were incubated top-down on a 25-μL drop of blocking buffer containing the primary antibody (rabbit polyclonal antibody generated against *S.* Typhimurium EAL) diluted to 1:50 overnight at room temperature. Grids were washed by dipping in a beaker containing 10 mL blocking buffer for 1 min and then incubated top-down on a 25-μL drop of blocking buffer for 15 min. Grids were then transferred top-down to a 25-μL drop of goat anti-rabbit IgG-gold antibody (Millipore Sigma) and incubated for 1 h at room temperature. A final wash was performed by dipping repeatedly in a beaker containing 10 mL blocking buffer for 0.5 min and then dipping repeatedly in a beaker containing 10 mL water for 0.5 min. Where indicated, grids were poststained by incubating them on a droplet of filtered 4% aqueous uranyl acetate for 20 min, washed thoroughly by dipping them into a beaker of deionized (DI) H_2_O 30 times, and then incubated on a drop of filtered DI H_2_O for 1 min. Negative controls were treated exclusively with secondary antibody. Imaging was performed using a JEOL 1011 transmission electron microscope (JEOL USA, Peabody, MA).

### Cobamide extraction and identification.

The isolation and identification of cobamides were performed as described previously (32). Briefly, 100 mL of M9 minimal medium containing ethanolamine (90 mM) as the sole source of carbon and energy and cobinamide (100 nM) was inoculated with 2.5% (vol/vol) of an LB liquid culture, and the culture was grown overnight at 37°C with shaking at 180 rpm. The following morning, cultures were harvested using a 5810 R centrifuge (Eppendorf) at 3,220 × *g* for 20 min. The cell pellet was resuspended in 10 mL buffer A (50 mM KH_2_PO_4_ [pH 6.5], 5 mM KCN) with 20 mL methanol and then incubated at 65°C for 2 h with shaking (220 rpm). Cell debris was removed by centrifugation at 30,000 × *g* in an Avanti JP20-XVI centrifuge equipped with a JA-25.50 rotor for 1 h. A SepPak C_18_ Plus cartridge (360 mg sorbent per cartridge, 55- to 105-μm particle size; Waters) was activated with methanol (10 mL) and water (10 mL). The supernatant was diluted 1:5 in water and passed over the activated SepPak cartridge. Cobamides bound to the SepPak cartridge were washed with water (20 mL) and eluted using methanol (100% [vol/vol]; 1.5 mL). Cobamides were dried overnight in a VacFuge Plus instrument (Eppendorf) and resuspended in buffer A (200 mL). KCN was added to a final concentration of 25 mM, and the mixture was incubated for 10 min in a sand bath kept at 90°C. Cobamides were then passed over a Spin-X centrifuge tube filter (Corning) for 5 min at 13,000 × *g*. Cleaned reaction mixtures were separated by RP-HPLC on a Shimadzu Prominence Ultra Fast Liquid Chromatography (UFLC) SPD-M30A instrument equipped with a Synergi 4μ Hydro-RP 80Å 150- by 4.6-mm column. The following method was used to separate cobamides. The column was equilibrated with 97% mobile phase A (MP A) (KH_2_PO_4_ [0.1 M] [pH 6.5], KCN [10 mM])–3% mobile phase B (MP B) (KH_2_PO_4_ [0.1 M] [pH 8], KCN [10 mM]-acetonitrile in a 1:1 ratio). Sample injection was followed by a 5-min linear gradient to 75% MP A–25% MP B, a 15-min linear gradient to 65% MP A–35% MP B, a 5-min isocratic step at 65% MP A–35% MP B, and a 2.5-min linear gradient to 100% MP B, concluding with a 5-min isocratic step at 100% MP B. Cobamides were identified by measuring the absorbance at 367 nm. Fractions containing cobamides were collected, desalted using a SepPak C_18_ cartridge, and dried overnight in a VacFuge Plus instrument. Cobamide fractions were resuspended in sterile water and sent for MALDI-TOF mass spectrometry analysis.
