# Supplementary material for: Acinetobacter baumannii Catabolizes Ethanolamine in the Absence of a Metabolosome and Converts Cobinamide into Adenosylated Cobamides
Source: mBio. 2022 Jul 26;13(4):e01793-22. doi: 10.1128/mbio.01793-22 (PMC9426561; doi:10.1128/mbio.01793-22)
Supplement: FIG S1 [file mbio.01793-22-s0001.pdf]

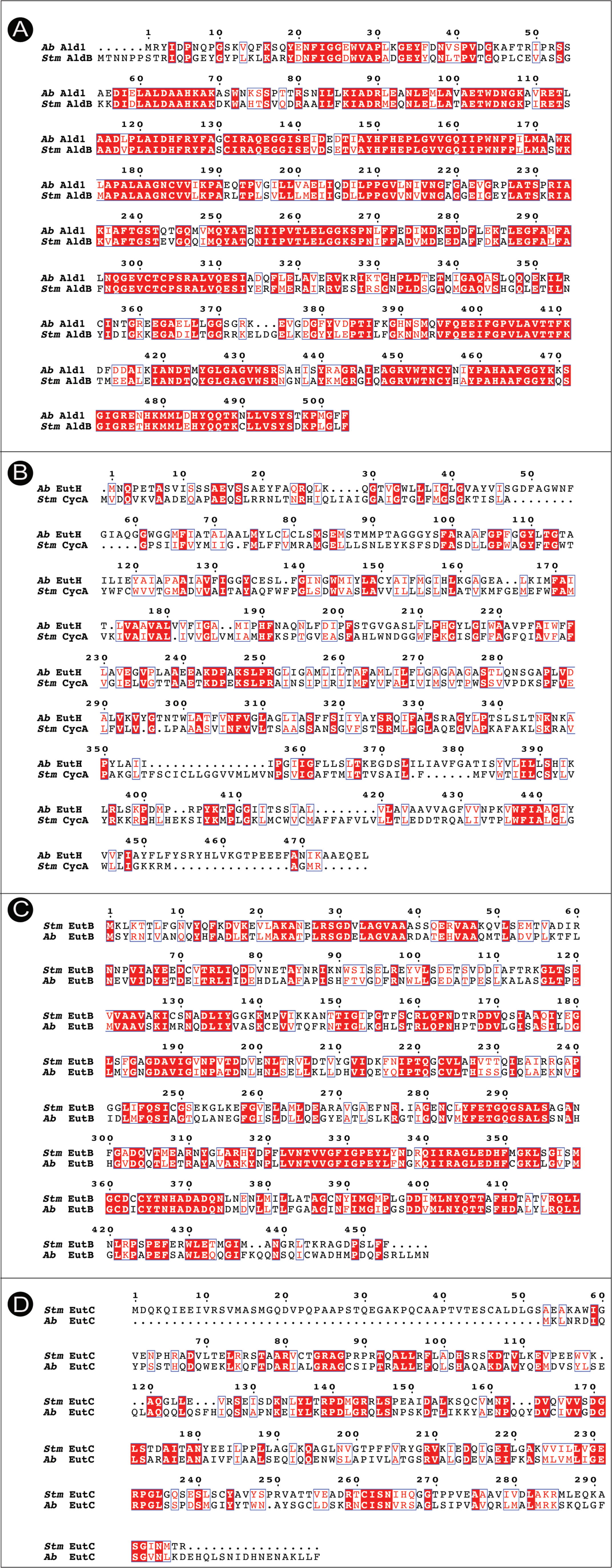
A
Ab Ald1 ......MRYIDPNQPGSKVQFKSQYENFIGGEWVAPLKGEYFDNVSPVDGKAFTRIPRSS
Stm AldB MTNNPPSTRIQPGEYGYPLKLKARYDNFIGGDWVAPADGEYYQNLTPVTGQPLCEVASSG
Ab Ald1 AEDIELALDAAHKAKASWNKSSPTTRSNILLKIADRLEANLEMLAVAETWDNGKAVRETL
Stm AldB KKDIDLALDAAHKAKDKWAHTSVQDRAAILFKIADRMEQNLELLATAETWDNGKPIRETS
Ab Ald1 AADLPLAIDHFRYFAGCIRAQEGGISEIDEDTIAYHFHEPLGVVGQIIPWNFPILMAAWK
Stm AldB AADVPLAIDHFRYFASCIRAQEGGISEVDSETVAYHFHEPLGVVGQIIPWNFPLLMASWK
Ab Ald1 LAPALAAGNCVVIKPAEQTPVGILLVAELIQDILPPGVLNIVNGFGAEVGRPLATSPRIA
Stm AldB MAPALAAGNCVVLKPARLTPLSVLLLMEIIGDLLPPGVVNVVNGAGGEIGEYLATSKRIA
Ab Ald1 KIAFTGSTQTGQMVMQYATENIIPVTLELGGKSPNLFFEDIMDKEDDFLEKTLEGFAMFA
Stm AldB KVAFTGSTEVGQQIMQYATQNIIPVTLELGGKSPNIFFADVMDEEDAFFDKALEGFALFA
Ab Ald1 LNQGEVCTCPSRALVQESIADQFLELAVERVKRIKTGHPLDTETMIGAQASLQQQEKILR
Stm AldB FNQGEVCTCPSRALVQESIYERFMERAIRRVESIRSGNPLDSGTQMGAQVSHGQLETILN
Ab Ald1 CINTGREEGAELLGGSGRK...EVGDGFYVDPTIFKGHNSMQVFQEEIFGPVLAVTTFK
Stm AldB YIDIGKKEGADILTGGRRKELDGELKEGYYLEPTILFGKNNMRVFQEEIFGPVLAVTTFK
Ab Ald1 DFDDAIKIANDTMYGLGAGVWSRSAHISYRAGRAIEAGRVWTNCYNIYPAHAAFGGYKKS
Stm AldB TMEEALEIANDTQYGLGAGVWSRNGNLAYKMGRGIQAGRVWTNCYHAYPAHAAFGGYKQS
Ab Ald1 GIGRENHKMMLDHYQQTKNLLVSYSTKPMGFF
Stm AldB GIGRETHKMMLEHYQQTKCLLVSYSDKPLGLF
B
Ab EutH .MNQPETASVISSSAEVSSAEYFAQRQLK....QGTVGWLLLIGLGVAYVISGDFAGWNF
Stm CycA MVDQVKVAADEQAPAEQSLRRNLTNRHIQLIAIGGAIGTGLFMGSGKTISLA........
Ab EutH GIAQGGWGGMFIATALAALMYLCLCLSMSEMSTMMPTAGGGYSFARAAFGPFGGYLTGTA
Stm CycA .....GPSIIFVYMIIG.FMLFFVMRAMGELLLSNLEYKSFSDFASDLLGPWAGYFTGWT
Ab EutH ILIEYAIAPAAIAVFIGGYCESL.FGINGWMIYLACYAIFMGIHLKGAGEA..LKIMFAI
Stm CycA YWFCWVVTGMADVVAITAYAQFWFPGLSDWVASLAVVILLLSLNLATVKMFGEMEFWFAM
Ab EutH T.LVAAVALVVFIGA..MIPHFNAQNLFDIPFSTGTVGASLFLPHGYLGIWAAVPFAIWFF
Stm CycA VKIVAIVAL.IVVGLVMIAMHFKSPTGVEASFAHLWNDGGWFPKGISGFFAGFQIAVFAF
Ab EutH LAVEGVPLAAEEAKDPAKSLPRGLIGAMLILTAFAMLILFLGAGAAGASTLQNSGAPLVD
Stm CycA VGIELVGTTAAETKDPEKSLPRAINSIPIRIIMFYVFALIVIMSVTPWSSVVPDKSPFVE
Ab EutH ALVKVYGTNTWLATFVNFVGLAGLIASFFSIIYAYSRQIFALSRAGYLPTSLSLTNKNKA
Stm CycA LFVLV.G.LPAAASVINFVVLTSAASSANSGVFSTSRMLFGLAQEGVAPKAFAKLSKRAV
Ab EutH PYLAII...............IPGIIGFLLSLTKEGDSLILIAVFGATISYVLILLSHIK
Stm CycA PAKGLTFSCICLLGGVVMLMVNPSVIGAFTMITTVSAIL.F......MFVWTIILCSYLV
Ab EutH LRLSKPDMP..RPYKTPGGIITSSIAL........VLAVAAVVAGFVVNPKVWFIAAGIY
Stm CycA YRKKRPHLHEKSIYKMPLGKLMCWVCMAFFAFVLVLLTLEDDTRQALIVTPLWFIALGLG
Ab EutH VVFIAYFLFYSRYHLVKGTPEEEFANIKAAEQEL
Stm CycA WLLIGKKRM...............AGMR......
C
Stm EutB MKLKTTLFGNVYQFKDVKEVLAKANELRSGDVLAGVAAASSQERVAAKQVLSEMTVADIR
Ab EutB MSYRNIVANQQYHFADLKTLMAKATPLRSGDELAGVAARDATEHVAAQMTLADVPLKTFL
Stm EutB NNPVIAYEEDCVTRLIQDDVNETAYNRIKNWSISELREYVLSDETSVDDIAFTRKGLTSE
Ab EutB NEVVIDYETDEITRLIIDEHDLAAFAPISHFTVGDFRNWLLGEDATPESLKALASGLTPE
Stm EutB VVAAVAKICSNADLIYGGKKMPVIKKANTTIGIPGTFSCRLQPNDTRDDVQSIAAQIYEG
Ab EutB MVAAVSKIMRNQDLIYVASKCEVVTQFRNTIGLKGHLSTRLQPNHPTDDVLGISASILDG
Stm EutB LSFGAGDAVIGVNPVTDDVENLTRVLDTVYGVIDKFNIPTQGCVLAHVTTQIEAIRRGAP
Ab EutB LMYGNGDAVIGINPATDNLHNLSELLKLLDHVIQEYQIPTQSCVLTHISSGIQLAEKNVP
Stm EutB GGLIFQSICGSEKGLKEFGVELAMLDEARAVGAEFNR.IAGENCLYFETGQGSALSAGAN
Ab EutB IDLMFQSIAGTQLANEGFGISLDLLQEGYEATLSLKRGTIGQNVMYFETGQGSALSSNAH
Stm EutB FGADQVTMEARNYGLARHYDPFLVNTVVGFIGPEYLYNDRQIIRAGLEDHFMGKLSGISM
Ab EutB HGVDQQTLETRAYAVARKYNPLLVNTVVGFIGPEYLFNGKQIIRAGLEDHFCGKLLGVPM
Stm EutB GCDCCYTNHADADQNLNENLMILLATAGCNYIMGMPLGDDIMLNYQTTAFHDTATVRQLL
Ab EutB GCDICYTNHADADQNDMDVLLTLFGAAGINFIMGIPGSDDVMLNYQTTSFHDALYLRQLL
Stm EutB NLRPSPEFERWLETMGIM..ANGRLTKRAGDPSLFF.....
Ab EutB GLKPAPEFSAWLEQQGIFKQQNSQICWADHMPDQFSRLLMN
D
Stm EutC MDQKQIEEIVRSVMASMGQDVPQPAAPSTQEGAKPQCAAPTVTESCALDLGSAEAKAWIG
Ab EutC ....MKLNRDIQ
Stm EutC VENPHRADVLTELRRSTAARVCTGRAGPRPRTQALLRFLADHSRSKDTVLKEVPEEWVK.
Ab EutC YPSSTHQDQWEKLKQFTDARIALGRAGCSIPTRALLEFQLSHAQAKDAVYQEMDVSYLSE
Stm EutC ..AQGLLE...VRSEISDKNLYLTRPDMGRRLSPEAIDALKSQCVMNP...DVQVVVSDG
Ab EutC QLAQQQLQSFHIQSNAPNKEIYLKRPDLGRQLSNPSKDTLIKKYAENPQQYDVCIVVGDG
Stm EutC LSTDAITANYEEILPPLLAGLKQAGLNVGTPFFVRYGRVKIEDQIGEILGAKVVILLVGE
Ab EutC LSARAIEANAIVFIAALSEQIQQENWSLAPIVLATGSRVALGDEVAEIFKASMLVMLIGE
Stm EutC RPGLGQSESLSCYAVYSPRVATTVEADRTCISNIHQGGTPPVEAAAVIVDLAKRMLEQKA
Ab EutC RPGLSSPDSMGIYYTWN.AYSGCLDSKRNCISNVRSAGLSIPVAVQRLMALMRKSKQLGF
Stm EutC SGINMTR.................
Ab EutC SGVNLKDEHQLSNIDHNENAKLLF
