# Supplementary material for: Acinetobacter baumannii Catabolizes Ethanolamine in the Absence of a Metabolosome and Converts Cobinamide into Adenosylated Cobamides
Source: mBio. 2022 Jul 26;13(4):e01793-22. doi: 10.1128/mbio.01793-22 (PMC9426561; doi:10.1128/mbio.01793-22)
Supplement: FIG S2 [file mbio.01793-22-s0002.pdf]

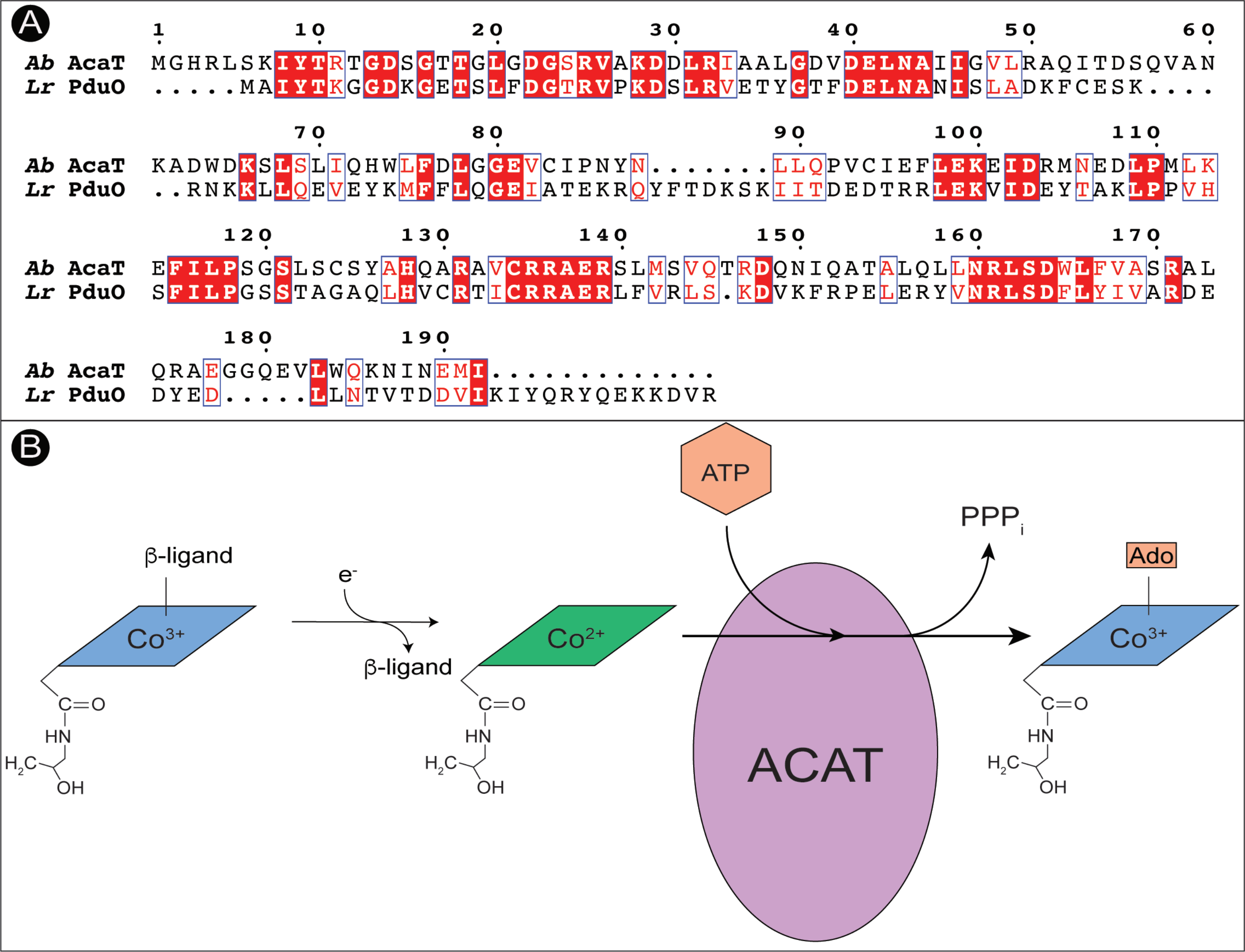
A
1 10 20 30 40 50 60
Ab AcaT MGHRLSKIYTRTGDSGTTGLGDGSRVAKDDLRIAALGDVDELNAIIGVLRAQITDSQVAN
Lr PduO .....MAIYTKGGDKGETSLFDGTRVPKDSLRVETYGTFDELNANISLADKFCESK....
70 80 90 100 110
Ab AcaT KADWDKSLSLIQHWLFDLGGEVCIPNYN.......LLQPVCIEFLEKEIDRMNEDLPMLK
Lr PduO ..RNKKLLQEVEYKMFFLQGEIATEKRQYFTDKSKIITDEDTRRLEKVIDEYTAKLPPVH
120 130 140 150 160 170
Ab AcaT EFILPSGSLSCSYAHQARAVCRRAERSLMSVQTRDQNIQATALQLLNRLSDWLFVASRAL
Lr PduO SFILPGSSTAGAQLHVCRTICRRAERLFVRLS.KDVKFRPELERYVNRLSDFLYIVARDE
180 190
Ab AcaT QRAEGGQEVLWQKNINEMI..............
Lr PduO DYED.....LLNTVTDDVIKIYQRYQEKKDVR
B
β-ligand
Co3+
e-
β-ligand
Co2+
ATP
PPPi
ACAT
Ado
Co3+
C=O
HN
H2C
OH
