# Supplementary material for: Acinetobacter baumannii Catabolizes Ethanolamine in the Absence of a Metabolosome and Converts Cobinamide into Adenosylated Cobamides
Source: mBio. 2022 Jul 26;13(4):e01793-22. doi: 10.1128/mbio.01793-22 (PMC9426561; doi:10.1128/mbio.01793-22)
Supplement: FIG S3 [file mbio.01793-22-s0003.pdf]

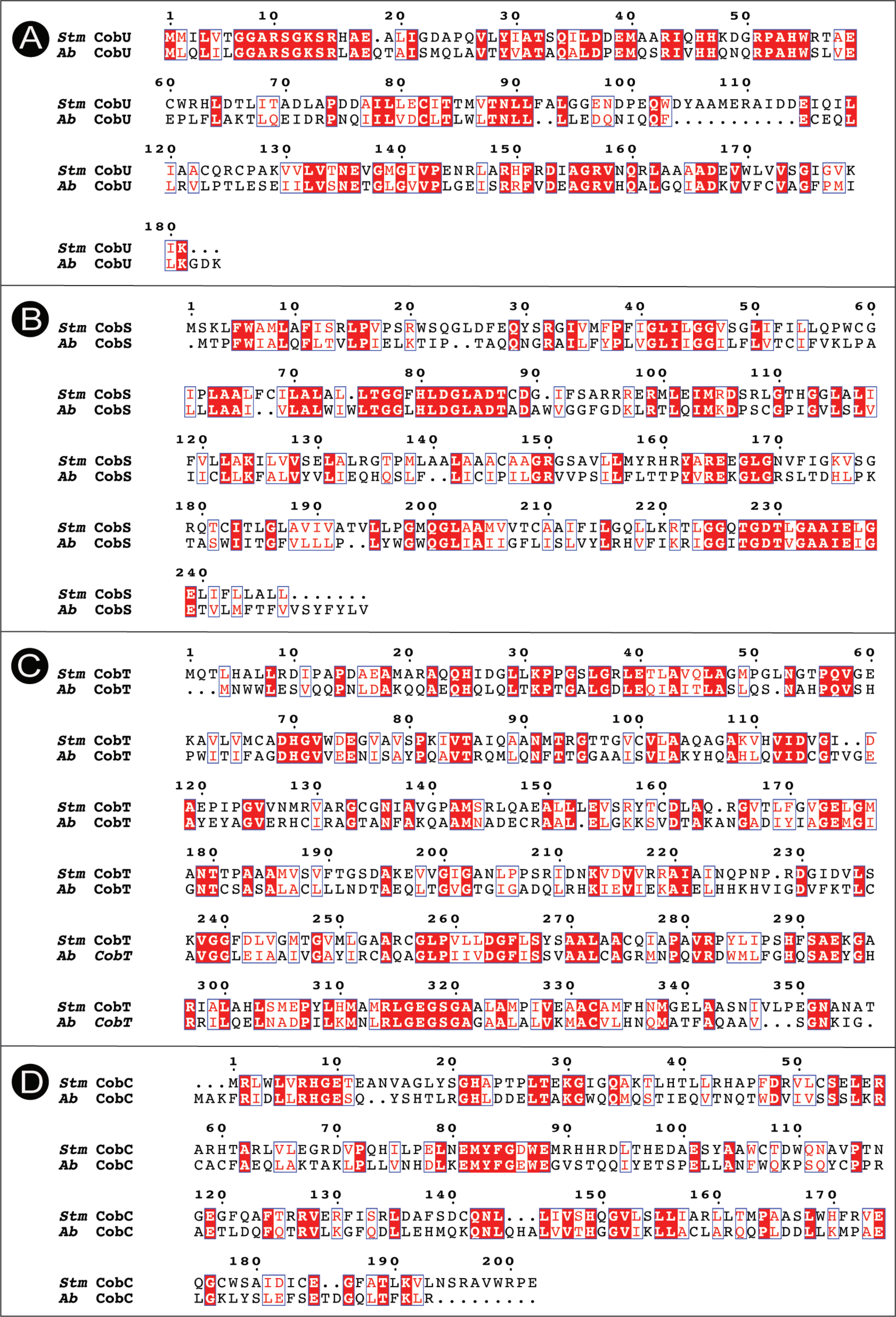
A
Stm CobU MMILVTGGARSGKSRHAE.ALIGDAPQVLYIATSQILDDEMAARIQHHKDGRPAHWRTAE
Ab CobU MLQLILGGARSGKSRLAEQTAISMQLAVTYVATAQALDPEMQSRIVHHQNQRPAHWSLVE
Stm CobU CWRHLDTLITADLAPDDAILLECITTMVTNLLFALGGENDPEQWDYAAMERAIDDEIQIL
Ab CobU EPLFLAKTLQEIDRPNQIILVDCLTLWLTNLL..LLEDQNIQQF...........ECEQL
Stm CobU IAACQRCPAKVVLVTNEVGMGIVPENRLARHFRDIAGRVNQRLAAAADEVWLVVSGIGVK
Ab CobU LRVLPTLESEIILVSNETGLGVVPLGEISRRFVDEAGRVHQALGQIADKVVFCVAGFPMI
Stm CobU IK...
Ab CobU LKGDK
B
Stm CobS MSKLFWAMLAFISRLPVPSRWSQGLDFEQYSRGIVMFPFIGLILGGVSGLIFILLQPWCG
Ab CobS .MTPFWIALQFLTVLPIELKTIP..TAQQNGRAILFYPLVGLIIGGILFLVTCIFVKLPA
Stm CobS IPLAALFCILALAL.LTGGFHLDGLADTCDG.IFSARRRERMLEIMRDSRLGTHGGLALI
Ab CobS LLLAAI..VLALWIWLTGGLHLDGLADTADAWVGGFGDKLRTLQIMKDPSCGPIGVLSLV
Stm CobS FVLLAKILVVSELALRGTPMLAALAAACAAGRGSAVLLMYRHRYAREEGLGNVFIGKVSG
Ab CobS IICLLKFALVYVLIEQHQSLF..LICIPILGRVVPSILFLTTPYVREKGLGRSLTDHLPK
Stm CobS RQTCITLGLAVIVATVLLPGMQGLAAMVVTCAAIFILGQLLKRTLGGQTGDTLGAAIELG
Ab CobS TASWIITGFVLLLP..LYWGWQGLIAIIGFLISLVYLRHVFIKRIGGITGDTVGAAIEIG
Stm CobS ELIFLLALL.......
Ab CobS ETVLMFTFVVSYFYLV
C
Stm CobT MQTLHALLRDIPAPDAEAMARAQQHIDGLLKPPGSLGRLETLAVQLAGMPGLNGTPQVGE
Ab CobT ...MNWWLESVQQPNLDAKQQAEQHQLQLTKPTGALGDLEQIAITLASLQS.NAHPQVSH
Stm CobT KAVLVMCADHGVWDEGVAVSPKIVTAIQAANMTRGTTGVCVLAAQAGAKVHVIDVGI..D
Ab CobT PWITIFAGDHGVVEENISAYPQAVTRQMLQNFTTGGAAISVIAKYHQAHLQVIDCGTVGE
Stm CobT AEPIPGVVNMRVARGCGNIAVGPAMSRLQAEALLLEVSRYTCDLAQ.RGVTLFGVGELGM
Ab CobT AYEYAGVERHCIRAGTANFAKQAAMNADECRAAL.ELGKKSVDTAKANGADIYIAGEMGI
Stm CobT ANTTPAAAMVSVFTGSDAKEVVGIGANLPPSRIDNKVDVVRRAIAINQPNP.RDGIDVLS
Ab CobT GNTCSASALACLLLNDTAEQLTGVGTGIGADQLRHKIEVIEKAIELHHKHVIGDVFKTLC
Stm CobT KVGGFDLVGMTGVMLGAARCGLPVLLDGFLSYSAALAACQIAPAVRPYLIPSHFSAEKGA
Ab CobT AVGGLEIAAIVGAYIRCAQAGLPIIVDGFISSVAALCAGRMNPQVRDWMLFGHQSAEYGH
Stm CobT RIALAHLSMEPYLHMAMRLGEGSGAALAMPIVEAACAMFHNMGELAASNIVLPEGNANAT
Ab CobT RRILQELNADPILKMNLRLGEGSGAGAALALVKMACVLHNQMATFAQAAV...SGNKIG.
D
Stm CobC ...MRLWLVRHGETEANVAGLYSGHAPTPLTEKGIGQAKTLHTLLRHAPFDRVLCSELER
Ab CobC MAKFRIDLLRHGESQ..YSHTLRGHLDDELTAKGWQQMQSTIEQVTNQTWDVIVSSSLKR
Stm CobC ARHTARLVLEGRDVPQHILPELNEMYFGDWEMRHHRDLTHEDAESYAAWCTDWQNAVPTN
Ab CobC CACFAEQLAKTAKLPLLVNHDLKEMYFGEWEGVSTQQIYETSPELLANFWQKPSQYCPPR
Stm CobC GEGFQAFTRRVERFISRLDAFSDCQNL...LIVSHQGVLSLLIARLLTMPAASLWHFRVE
Ab CobC AETLDQFQTRVLKGFQDLLEHMQKQNLQHALVVTHGGVIKLLACLARQQPLDDLLKMPAE
Stm CobC QGCWSAIDICE..GFATLKVLNSRAVWRPE
Ab CobC LGKLYSLEFSETDGQLTFKLR.........
