# Supplementary figures and images for: Acinetobacter baumannii Catabolizes Ethanolamine in the Absence of a Metabolosome and Converts Cobinamide into Adenosylated Cobamides
Source: mBio. 2022 Jul 26;13(4):e01793-22. doi: 10.1128/mbio.01793-22 (PMC9426561; doi:10.1128/mbio.01793-22)

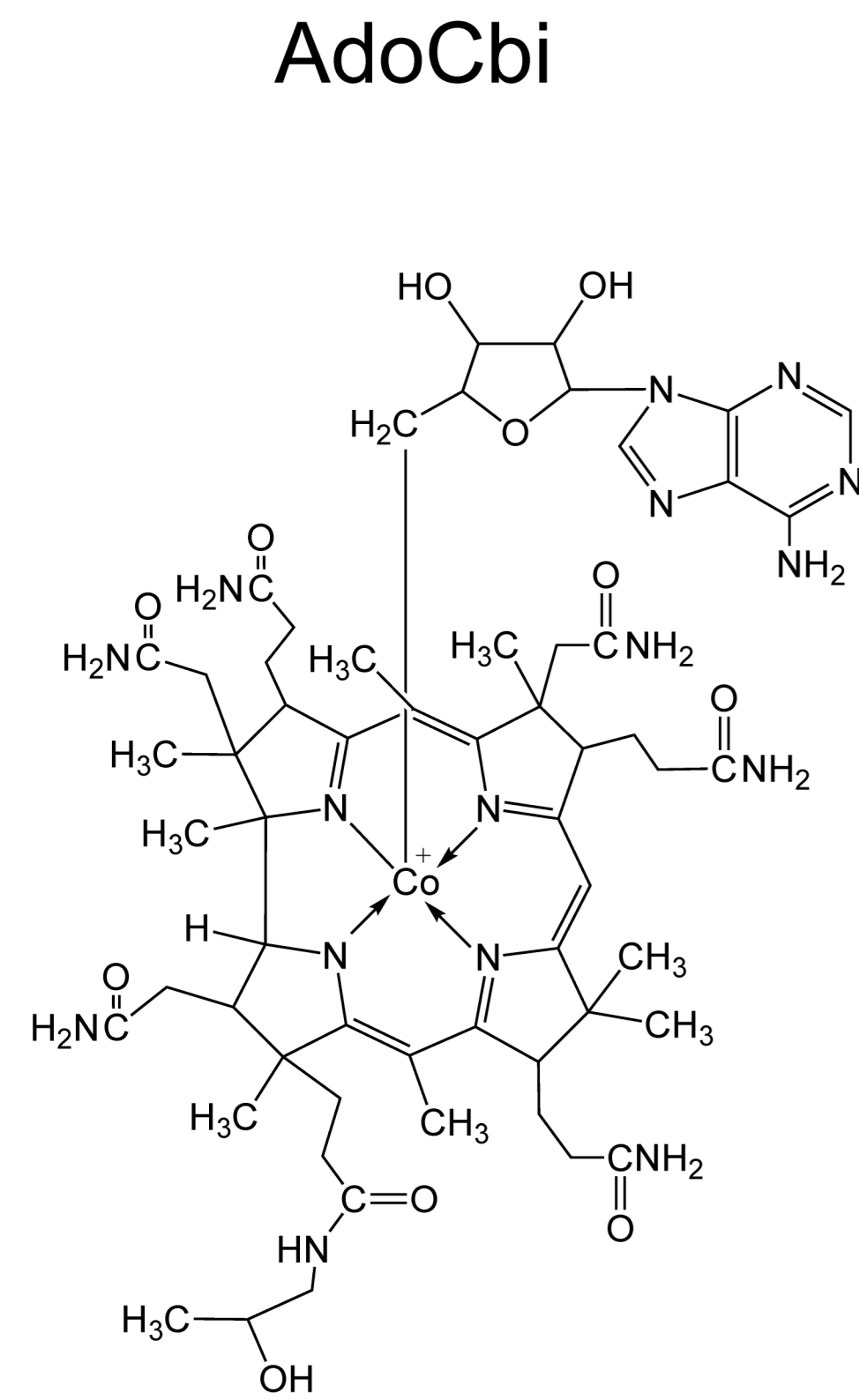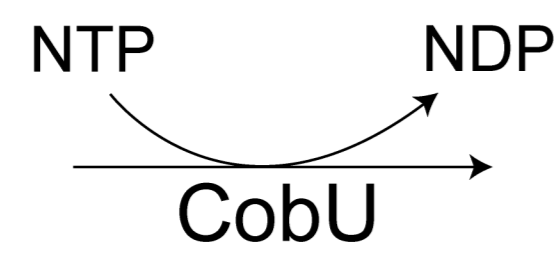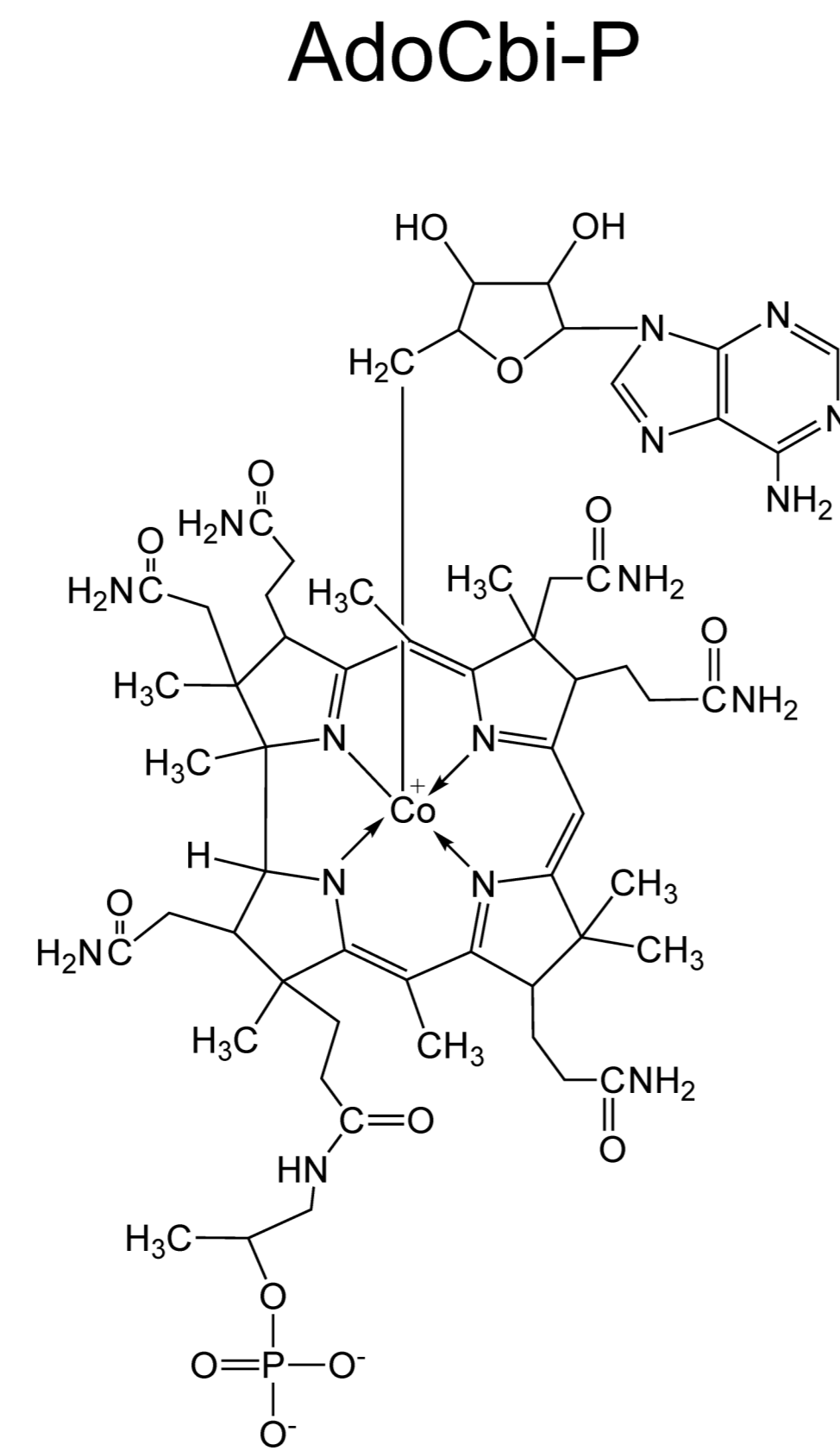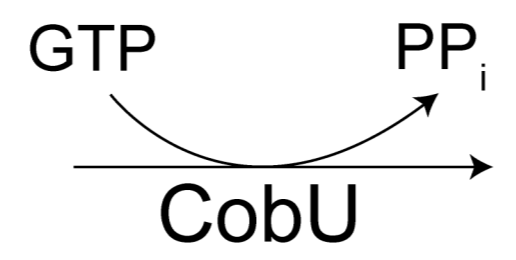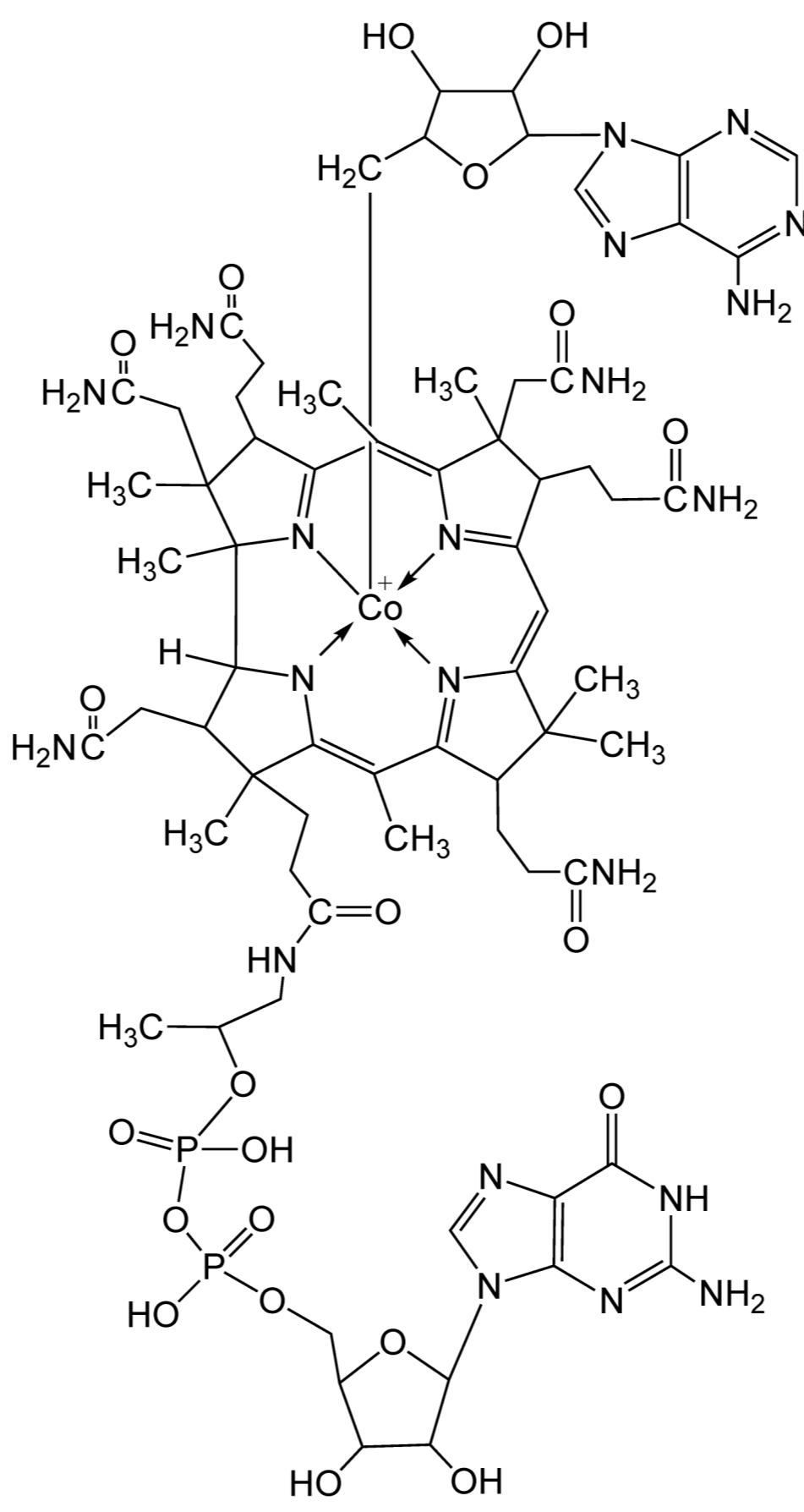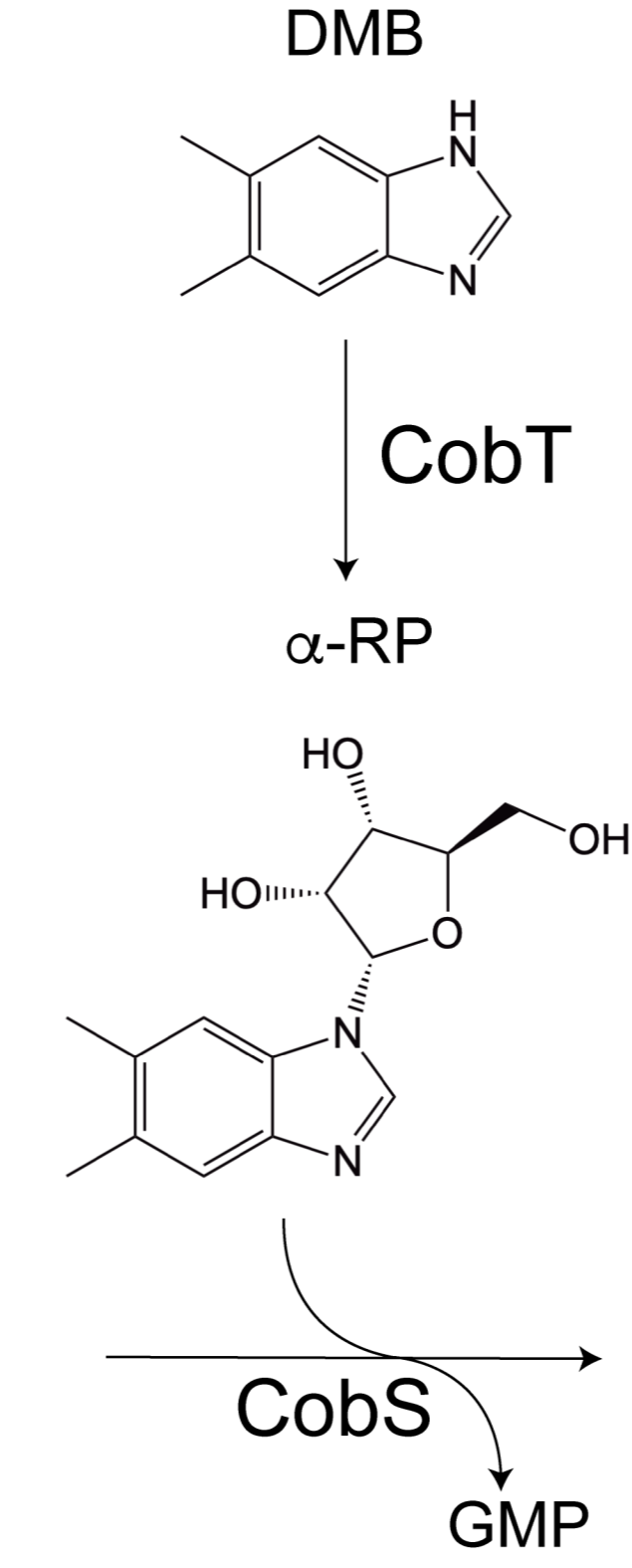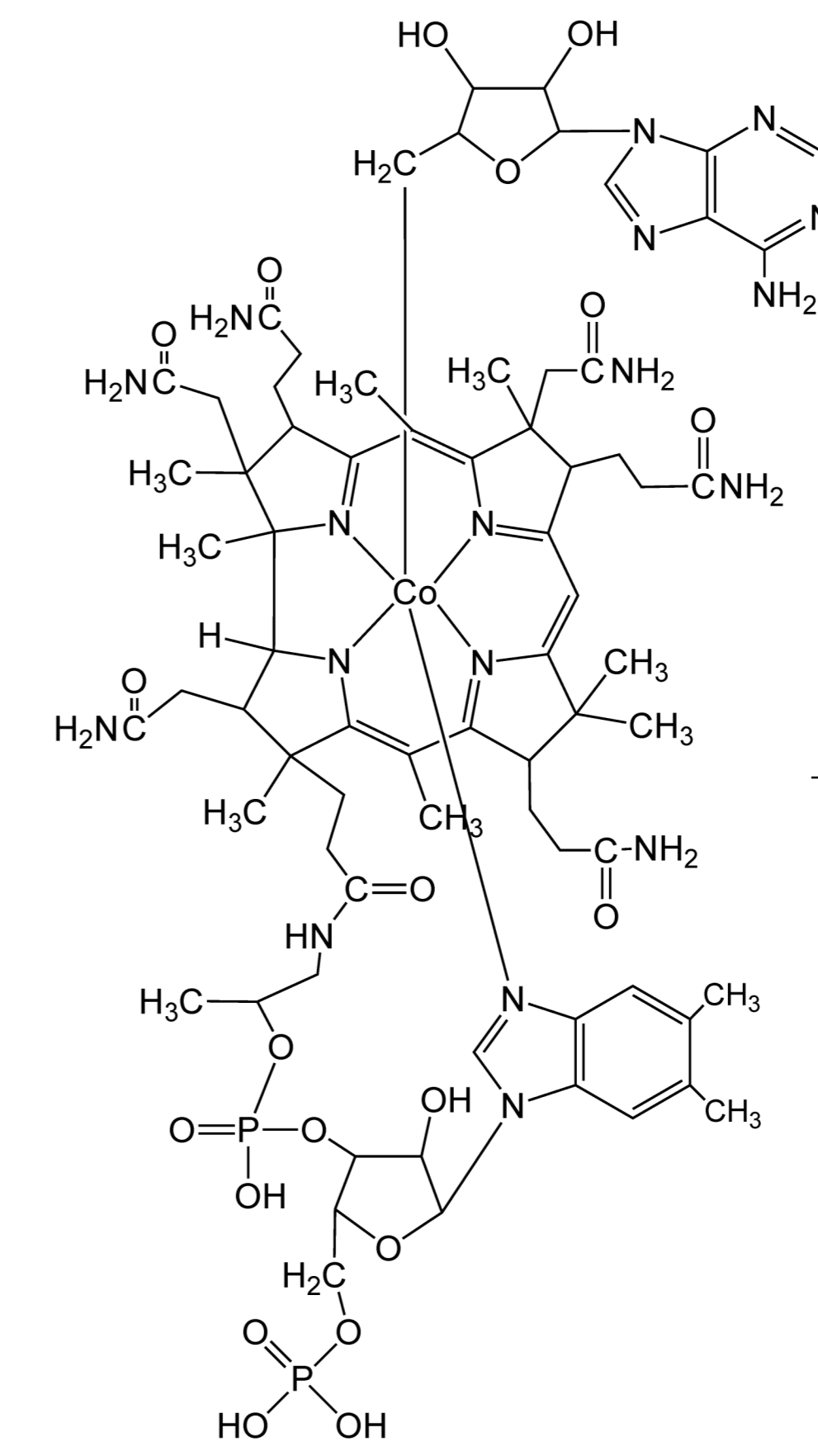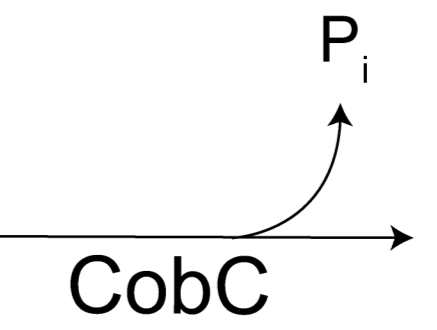

Supplement: FIG S4 [file mbio.01793-22-s0004.pdf]

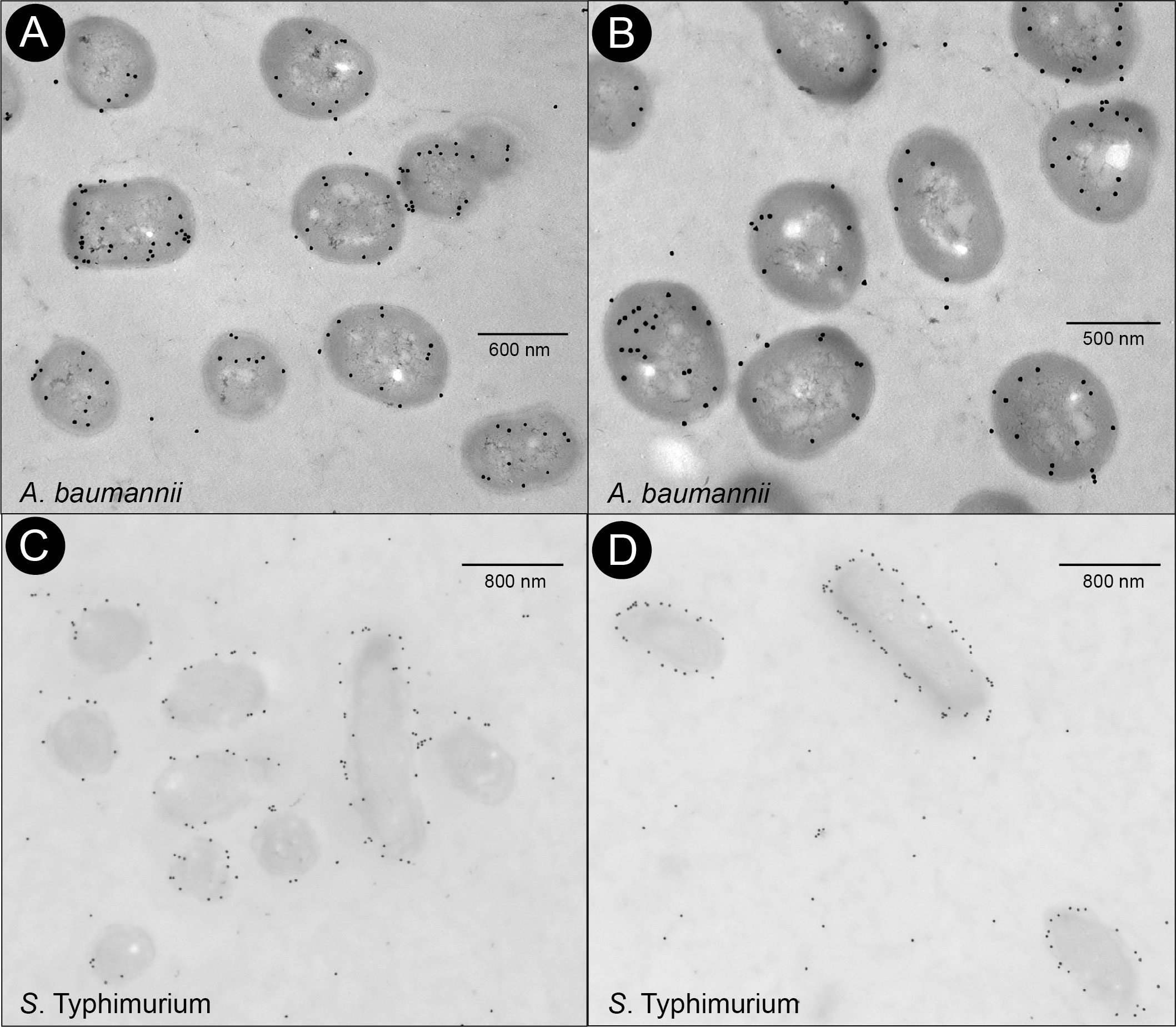

Supplement: FIG S6 [file mbio.01793-22-s0006.jpg]

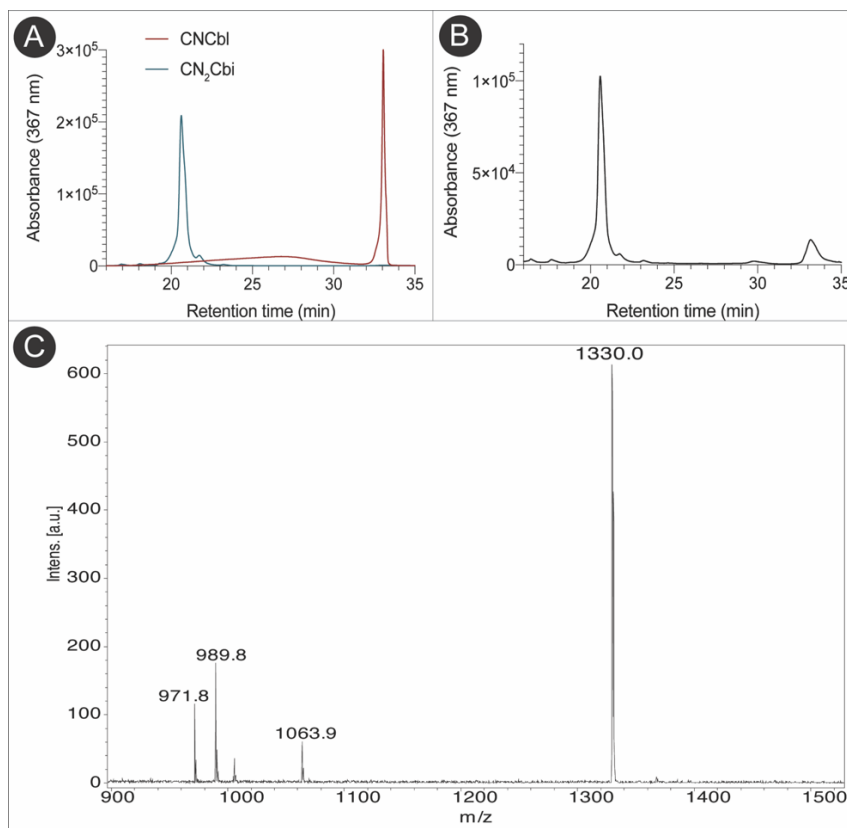

Supplement: FIG S8 [file mbio.01793-22-s0008.pdf]
