# Supplementary material for: Acinetobacter baumannii Catabolizes Ethanolamine in the Absence of a Metabolosome and Converts Cobinamide into Adenosylated Cobamides
Source: mBio. 2022 Jul 26;13(4):e01793-22. doi: 10.1128/mbio.01793-22 (PMC9426561; doi:10.1128/mbio.01793-22)
Supplement: FIG S5 [file mbio.01793-22-s0005.pdf]

TAAATAAAAGCTCTGTACACGACAAATTTCACAGAACCCTTATCCTATCAGGGTTCTGCCTTCTTAAAATTGCCAAAATTT
CCTTAAACTCTTCTTTTTTCCCAAAACCAATTAAACGCTGAATCGCCATTTGAACATAGTCTAAACCATAGCGAAATAAAC
TCATTGAGAGTCGTCCATGCTTCTTTATTTTTATCGCTTTTTTTTGATCATGTTGCCATTCACCCGTTAAGTAACACCAAC
AGAAGCTTATAGCTAACACCGCAATCAATTTTTTCACTCGTCTAGGGTCTGTCAAGCGCGTATTTTCAAGATTAAACCCGC
GTCCTTTGAGACAACTGAATAAGGTTTCAATTTCCCAGCGTAATGCATAATCCTGAATAGCATTGGCATTAAACTGAGGAG
AAACGACGAGTAAAAGCTCTCCATTTTCTAACTGTAGTGCACTTATATATAGTTTCACCCGACCAACCAAAATCCGTCGTT
TACGACATTCAATTTGACCAACTTTAAGATGGCGAAATAAATCACTAATTTTATGATTCTTTCCTAAATGATTGGTGACAA
TGAAGTTTTTTTAACACGAATGCAGAAGTTGATGTCTTGTTCAATTAACCATGTAAACCACTGCTCACCGATAAACTCTCT
GTCTGCGAACACATTCACAATACGGTCTTTACCAAAAATGGCTATAAAGCGTTGAATCAAAGCAATACGCTCTTTCGTATC
TGAATTTCCACGTTTATTAAGCAATGTCCAAAGGATAGGTATCGCTATTCCACGATAAACGATTGCGAGCATCAGGATATT
AATATTTCGTTTTCCCATTTCCAATTGGTTCTATCTAAAGTCAGTTGCACTTGGTCGAATGAAAACATATTGAAAATCAA
CTGAGAAATTTGACGATAATCAAAATACTGACCTGCAAAGAAGCGCTGCATACGTCGATAAAATGATTGTGGTAAGCACTT
GATGGGCAAGGCTTTAGATGCAGAAGAAAGATTACATGTTTGCTTTAAAATAATCACAAGCATGATGAGCGCAAAGCACTT
TAAATGTGACTTGTTCCATTTTAGATATTTGTTTAAGATAAGATATAACTCATTGAGATGTGTCATAGTATTCGTCGTTAG
AAAACAATTATTATGACATTATTTCAATGAGTTATCTATTTTTATCGTGTACAGAGAAATAAAAGGATGTAACTATG
