# Supplementary material for: Acinetobacter baumannii Catabolizes Ethanolamine in the Absence of a Metabolosome and Converts Cobinamide into Adenosylated Cobamides
Source: mBio. 2022 Jul 26;13(4):e01793-22. doi: 10.1128/mbio.01793-22 (PMC9426561; doi:10.1128/mbio.01793-22)
Supplement: TABLE S1 [file mbio.01793-22-s0009.pdf]

Table S1. Strains and plasmids used in this study[a]		
**Strain**	**Genotype**	**Reference/source**
***Salmonella enterica* subsp. *enterica* sv. Typhimurium str. LT2 strains**		
JE6583	*metE205 araB9*	Lab collection
JE7088	Δ*metE2702 araB9*	Lab collection
**Derivatives of strain JE6583**		
JE8094	Δ*eutBC1156*	Lab collection
JE8248	Δ*cobS1313*	Lab collection
JE8249	Δ*cobU1315*	Lab collection
JE8268	Δ*cobU1315* Δ*ycfN112*	Lab collection
JE15888	Δ*cobS1313* / pCOBS69 (*Se*CobS)	Lab collection
JE16852	Δ*cobS1313 /* pBAD24	Lab collection
JE22175	Δ*eutSPQTDMNEJGHABCLK* (*zfa3648**Tn*10***zfa3649*)	Lab collection
JE23613	Δ*pduO522* Δ*cobA1465* Δ*eutT1141*	Lab collection
JE25835	Δ*pduO522* Δ*cobA1465* Δ*eutT1141* / pCobA70 (*Se*CobA)	
JE25836	Δ*pduO522* Δ*cobA1465* Δ*eutT1141* / pAbAcaT1	
JE25837	Δ*pduO522* Δ*cobA1465* Δ*eutT1141* / pBAD24	
JE25948	Δ*cobS1313* / pAbCobS1	
JE25949	Δ*cobU1315* / pCV1	
JE25950	Δ*cobU1315* Δ*ycfN112* / pAbCobU1	
JE25951	Δ*cobU1315* Δ*ycfN112* / pCV1	
JE25952	Δ*cobU1315* Δ*ycfN112* / pCobU27 (*Se*CobU)	
JE26300	Δ*eutBC1156* / pCV1	
JE26301	Δ*eutBC1156* / pEut269 (*Se*EutBC)	
JE26302	Δ*eutBC1156* / pAbEutBC1	
JE26303	Δ*eutABC1157* / pCV1	
JE26304	Δ*eutABC1157* / pEut269 (*Se*EutBC)	
JE26305	Δ*eutABC1157* / pAbEutBC1	
JE26306	Δ*eutSPQTDMNEJGHABCLK* (*zfa3648**Tn*10***zfa3649*) / pCV1	
JE26307	Δ*eutSPQTDMNEJGHABCLK* (*zfa3648**Tn*10***zfa3649*) / pEut269 (*Se*EutBC)	
JE26308	Δ*eutSPQTDMNEJGHABCLK* (*zfa3648**Tn*10***zfa3649*) / pAbEutBC1	
JE7088	Δ*metE2702 araB9*	
**Derivatives of strain JE7088**		
JE20198	Δ*cobB1375 cobT1379*::*kan*$^+$	Lab collection
JE26016	Δ*cobB1375 cobT1379*::*kan*$^+$ / pCV1	
JE26017	Δ*cobB1375 cobT1379*::*kan*$^+$ / pCobT140 (*Se*CobT)	
JE26018	Δ*cobB1375 cobT1379*::*kan*$^+$ / pAbCobT1	
**Derivatives of *Acinetobacter baumannii* str. 17978**		
JE26013	IS*Aba1* insertion (3,825,518-3,826,6706)	
JE26441	Δ*eutBC* / pAbEutBC3	
JE26442	/ pMMB67EH	
**Plasmid**	**Description**	**Reference/source**
pMMB67EHKn	*kan*$^+$	(1)
pKD4	*kan*$^+$	(2)

pRecABtet	*recET*+ cloned into pMMB67EH, *tet*+	(3)
pFLPtet	FLP recombinase gene cloned into pMMB67EH, *tet*+	(3)
pBAD24	Complementation vector P_{araBAD} *bla*+	(4)
pCV1	Complementation vector P_{araBAD} *bla*+	(5)
pCobU27	*S.* Typhimuirum *cobU*+ cloned into pBAD24	Lab collection
pCobS69	*S.* Typhimuirum *cobS*+ cloned into pBAD24	Lab collection
pCobT140	*S.* Typhimuirum *cobT*+ cloned into pBAD24	Lab collection
pCobA70	*S.* Typhimuirum *cobA*+ cloned into pBAD24	Lab collection
pEut269	*S.* Typhimuirum *eutBC*+ cloned into pBAD24	Lab collection
pAbCobU1	*A. baumannii cobU*+ cloned into pCV1	
pAbCobS1	*A. baumannii cobS*+ cloned into pCV1	
pAbCobT1	*A. baumannii cobT*+ cloned into pCV1	
pAbAcaT1	*A. baumannii acaT*+ cloned into pCV1	
pAbEutBC1	*A. baumannii eutBC*+ cloned into pCV1	
pAbEutBC3	*A baumannii eutBC*+ cloned into pMMB67EH	

[a]All strains and plasmids were constructed during the course of this work unless otherwise stated.

Supplemental references

1. Boll JM, Crofts AA, Peters K, Cattoir V, Vollmer W, Davies BW, Trent MS. 2016. A penicillin-binding protein inhibits selection of colistin-resistant, lipooligosaccharide-deficient *Acinetobacter baumannii*. Proc Nat Acad Sci U S A 113:E6228-E6237.
2. Datsenko KA, Wanner BL. 2000. One-step inactivation of chromosomal genes in *Escherichia coli* K-12 using PCR products. Proc Natl Acad Sci U S A 97:6640-6645.
3. Tucker AT, Nowicki EM, Boll JM, Knauf GA, Burdis NC, Trent MS, Davies BW. 2014. Defining gene-phenotype relationships in *Acinetobacter baumannii* through one-step chromosomal gene inactivation. MBio 5:e01313-14.
4. Cronan JE. 2006. A family of arabinose-inducible *Escherichia coli* expression vectors having pBR322 copy control. Plasmid 55:152-157.
5. VanDrisse CM, Escalante-Semerena JC. 2016. New high-cloning-efficiency vectors for complementation studies and recombinant protein overproduction in *Escherichia coli* and *Salmonella enterica*. Plasmid 86:1-6.
