# Supplementary material for: Acinetobacter baumannii Catabolizes Ethanolamine in the Absence of a Metabolosome and Converts Cobinamide into Adenosylated Cobamides
Source: mBio. 2022 Jul 26;13(4):e01793-22. doi: 10.1128/mbio.01793-22 (PMC9426561; doi:10.1128/mbio.01793-22)
Supplement: TABLE S2 [file mbio.01793-22-s0010.pdf]

Table S2. Primers used in this study[a]	
**Primer name**	**Primer sequence (5’ → 3’)**
5’ *AbeutB* deletion	ACCAGAAGAAGAGTTTGCAAATATTAAAGCCGCAGAACAAGA ACTATAAATAAAAGGATGTAACTATGAGTTATCGCAATATTGTC GCCAATCAACAGTATCACTTTGCTGACCTAAAAACGTTA GTGT AGGCTGGAGCTGCTTC
3’ *AbeutC* deletion	AAGATAATCTCGTTACGCATAAATATTCTGTAGGGAAGAAATA TCTACGTGTAACCTGCTATTTAAAATAAAAGCTTTGCGTTTTCA TTATGGTCGATATTTGAAAGTTGATGTTCATCTTTTAA CATATGAATATCCTCCTTAG
5’ *AbcobU* pCV1	NNGCTCTTCNTTCATGTTGCAATtgaTTTTAGG
3’ *AbcobU* pCV1	NNGCTCTTCNTTATTACTTATCACCTTTTAAAATC
5’ *AbcobS* pCV1	NNGCTCTTCNTTCATGACACCTTTTTGGATAGC
3’ *AbcobS* pCV1	NNGCTCTTCNTTATCAAACTAAATAAAAATAACTTACGAC
5’ *AbcobT* pCV1	NNGCTCTTCNTTCATGAACTGGTGGTTAGAATC
5’ *AbcobT* pCV1	NNGCTCTTCNTTATTAGCCAATTTTATTCCCG
5’ *AbacaT* pCV1	NNGCTCTTCNTTCatgGGACACCGTTTAAGCAAAATC
3’ *AbacaT* pCV1	NNGCTCTTCNTTAttaAATCATCTCATTAATATTTTTTTG
5’ *AbeutB* EcoRI	NNN GAATTC atgAGTTATCGCAATATTGTCGCC
5’ *AbeutC* SalI	NNNgtcgacTTAAAATAAAAGCTTTGCG
5’ *AbeutB* qPCR	ACTTCCTTCCATGATGCCCT
3’ *AbeutB* qPCR	TGAGCAGACGAGAGAATTGGT
5’ *AbeutC* qPCR	GAACGCCCTGGTTTAAGCTC
3’ *AbeutC* qPCR	TCACTCCTGAAAAGCCAAGC
5’ *Ab*16S qPCR	TCTGCAACTCGACTCCATGA
3’ *Ab*16S qPCR	ACGGCTACCTTGTTACGACT
5’ *Abald1* qPCR	GGTTTAGGTGCAGGTGTATGGT
3’ *Abald1* qPCR	AGCATGCGCAGGGTAGATG
5’ *Abald1-eutH*	GGTGGCTACAAGAAGTCTGGTATTG
3’ *Abald1-eutH*	GCTACGCCTAAACCAATCAGG
5’ *AbeutH-eutB*	GCAGTCGCTGCTGTGGTC
3’ *AbeutH-eutB*	CGCAACATGTTCGGTTGC
5’ *AbeutB-eutC*	CCCGCTCCTGAGTTCTCC
3’ *AbeutB-eutC*	GCTAATTGCTCCGAGAGGTAG
5’ *AbeutB* flanking check	NNNGCCGCTATCATTTAGTG
3’ *AbeutC* flanking check	NNNCGTCAATACCTGAAAATTGC

[a]All primers were synthesized by Integrated DNA Technologies (Coralville, IA)
